# A wheat NAC interacts with an orphan protein and enhances resistance to Fusarium head blight disease

**DOI:** 10.1111/pbi.13105

**Published:** 2019-04-29

**Authors:** Alexandre Perochon, Amal Kahla, Monika Vranić, Jianguang Jia, Keshav B. Malla, Melanie Craze, Emma Wallington, Fiona M. Doohan

**Affiliations:** ^1^ UCD School of Biology and Environmental Science and Earth Institute College of Science University College Dublin Belfield, Dublin 4 Ireland; ^2^ The John Bingham Laboratory NIAB Cambridge UK

**Keywords:** Deoxynivalenol, *Fusarium graminearum*, Fusarium head blight, NAC, orphan gene, SnRK1, transcription factor, *Triticum aestivum*, wheat

## Abstract

Taxonomically‐restricted orphan genes play an important role in environmental adaptation, as recently demonstrated by the fact that the *Pooideae*‐specific orphan *TaFROG* (*Triticum aestivum* Fusarium Resistance Orphan Gene) enhanced wheat resistance to the economically devastating Fusarium head blight (FHB) disease. Like most orphan genes, little is known about the cellular function of the encoded protein TaFROG, other than it interacts with the central stress regulator TaSnRK1α. Here, we functionally characterized a wheat (*T. aestivum*) NAC–like transcription factor TaNACL‐D1 that interacts with TaFROG and investigated its’ role in FHB using studies to assess motif analyses, yeast transactivation, protein‐protein interaction, gene expression and the disease response of wheat lines overexpressing *TaNACL‐D1*. TaNACL‐D1 is a *Poaceae*‐divergent NAC transcription factor that encodes a *Triticeae*‐specific protein C‐terminal region with transcriptional activity and a nuclear localisation signal. The TaNACL‐D1/TaFROG interaction was detected in yeast and confirmed *in planta*, within the nucleus. Analysis of multi‐protein interactions indicated that TaFROG could form simultaneously distinct protein complexes with TaNACL‐D1 and TaSnRK1α *in planta*. *TaNACL‐D1* and *TaFROG* are co‐expressed as an early response to both the causal fungal agent of FHB,* Fusarium graminearum* and its virulence factor deoxynivalenol (DON). Wheat lines overexpressing *TaNACL‐D1* were more resistant to FHB disease than wild type plants. Thus, we conclude that the orphan protein TaFROG interacts with TaNACL‐D1, a NAC transcription factor that forms part of the disease response evolved within the *Triticeae*.

## Introduction

Advances in genome sequencing technologies over the last decade have exponentially increased the availability of whole genomes in all different kingdoms, including plants and animals. This revealed that a significant portion of eukaryotic genomes encodes orphan genes (or taxonomically restricted genes). These genes are phylogenetically restricted and do not encode any previously identified protein domains (Khalturin *et al*., 2009). Despite the fact that orphan genes can represent about 10–20% of the genes encoded by eukaryotic genomes (Khalturin *et al*., 2009), their functions remain largely unknown (Arendsee *et al*., 2014). There is evidence that the great majority are transcribed and encode proteins, but their functional relevance still needs to be determined (McLysaght and Hurst, 2016; Prabh and Rodelsperger, 2016). However, there is evidence that some orphan genes play crucial roles in the survival of organisms, their knockdown resulting in lethality (Chen *et al*., 2010; Reinhardt *et al*., 2013). Orphan genes play a role in lineage‐specific traits during developmental processes, such as the formation of pre‐axial digits in Salamander (Kumar *et al*., 2015) or the creation of cnidocytes and the generation of morphological diversity in *Hydra* (Khalturin *et al*., 2009; Kumar *et al*., 2015). Orphan genes are often differentially expressed in response to biotic and abiotic stress, as seen across different species including Arabidopsis (*Arabidopsis thaliana*), rice (*Oryza sativa*) and the microcrustacean *Daphnia pulex* (Colbourne *et al*., 2011; Donoghue *et al*., 2011; Guo *et al*., 2007). Relatively few plant orphan genes have been studied in depth. Recent studies revealed that orphan genes are important players in key agronomic traits, including *Ms2* that confers male sterility in wheat (Ni *et al*., 2017), *QQS* (Qua‐Quine Starch) that regulates carbon and nitrogen partitioning across species (Li *et al*., 2015) and *TaFROG* (*Triticum aestivum Fusarium* Resistance Orphan Gene) that enhances wheat resistance to disease (Perochon *et al*., 2015).

The lack of characterized domains raises the question as to how orphan proteins function? One answer may come from the fact that they are intrinsically disordered proteins (IDPs) (Schmitz and Bornberg‐Bauer, 2017; Wilson *et al*., 2017). IDPs are keys players in cellular signaling, interactions or by functioning as hubs in signaling networks (Wright and Dyson, 2015). Because IDPs have unstructured properties, they often function through protein‐protein interactions (Dunker *et al*., 2005; Tompa *et al*., 2015). Thus, the identification of the key protein interactors will advance our understanding of the mechanisms through which orphan proteins operate. Previously, we characterized the orphan protein TaFROG and demonstrated its role in the resistance of wheat to Fusarium head blight (FHB) disease caused by the mycotoxigenic fungus *Fusarium graminearum*. We found that TaFROG is an IDP that can interact with the Sucrose Non‐Fermenting1‐Related Kinase1 (SnRK1); SnRK1 is a key signaling protein, and is the orthologue of the yeast Sucrose Non‐Fermenting1 (SNF1) and the mammalian AMP‐activated protein kinase (AMPK) (Perochon *et al*., 2015). SnRK1 was initially identified as an interactor via a yeast two‐hybrid screen of a wheat cDNA library using TaFROG as bait; other potential TaFROG interactors identified in the same screen included a histone‐binding protein and a NAC [No apical meristem (NAM), *Arabidopsis* transcription activation factor (ATAF), Cup‐shaped cotyledon (CUC)] transcription factor. Thus, it seems possible that TaFROG interacts with functionally diverse proteins.

The NAC protein identified within the screen for TaFROG interactors belongs to one of the largest families of plant transcription factors. Typically, NAC proteins are characterized by a conserved N‐terminal NAC domain and a poorly conserved C‐terminal region (Kikuchi *et al*., 2000; Olsen *et al*., 2005). NAC transcription factors are known to be involved in different processes, including developmental programs (Olsen *et al*., 2005), senescence (Kim *et al*., 2016), biotic and abiotic stress responses (Puranik *et al*., 2012). Recently, wheat was estimated to encode 453 NACs based on The Genome Analysis Centre (TGAC) gene models TGACv1 (Borrill *et al*., 2017). Very little is known about their physiological functions and our knowledge about their role in biotic stress is very limited. In wheat, NACs have been shown to play a negative role regulating the defense against stripe rust disease (Bing *et al*., 2018; Feng *et al*., 2014; Wang *et al*., 2015; Xia *et al*., 2010).

In this study, we demonstrate that a NAC protein can interact with the orphan protein TaFROG and that it positively enhances disease resistance in wheat. Analysis of the protein sequence demonstrated that this NAC was *Poaceae*‐specific and located on the wheat chromosome 5D and has a divergent NAC domain, therefore, we designate it as *T. aestivum* NAC‐like D1 (*TaNACL‐D1*). We showed that TaNACL‐D1 is localized in the nucleus and that it has transactivation activity. Furthermore, our results illustrated that *TaNACL‐D1* is co‐expressed with *TaFROG* during the development of FHB disease caused by *F. graminearum*. TaNACL‐D1 protein interacted with TaFROG in yeast and *in planta* and formed a protein complex that was subcellular distinct from the TaNACL‐D1‐TaSnRK1α complex. Finally, we present evidence that *TaNACL‐D1* contributes to FHB resistance in wheat, as also shown in a previous study for *TaFROG* (Perochon *et al*., 2015).

## Results

### TaNACL‐D1 is a divergent NAC transcription factor

A wheat NAC‐like gene was identified during a yeast two‐hybrid screen conducted to identify interactors of *Fusarium* resistance orphan protein TaFROG (Perochon *et al*., 2015). The *T. aestivum* NAC‐like transcription factor gene was cloned and sequenced from cv CM82036 and located to chromosome 5D (*TaNACL‐D1*). The deduced coding sequence shares 100% identity with gene *TraesCS5D02G111300* located on chromosome 5D of bread wheat cv Chinese Spring (IWGSC RefSeq v1.1). *TaNACL‐D1* shares 95 and 94% identity with the homeologous genes on chromosomes 5A (*TraesCS5A02G099000*) and 5B (*TraesCS5B02G104200*), respectively of the cv Chinese Spring genome and these were thereafter named *TaNACL‐A1* and *TaNACL‐B1,* respectively.

**Figure 1:**
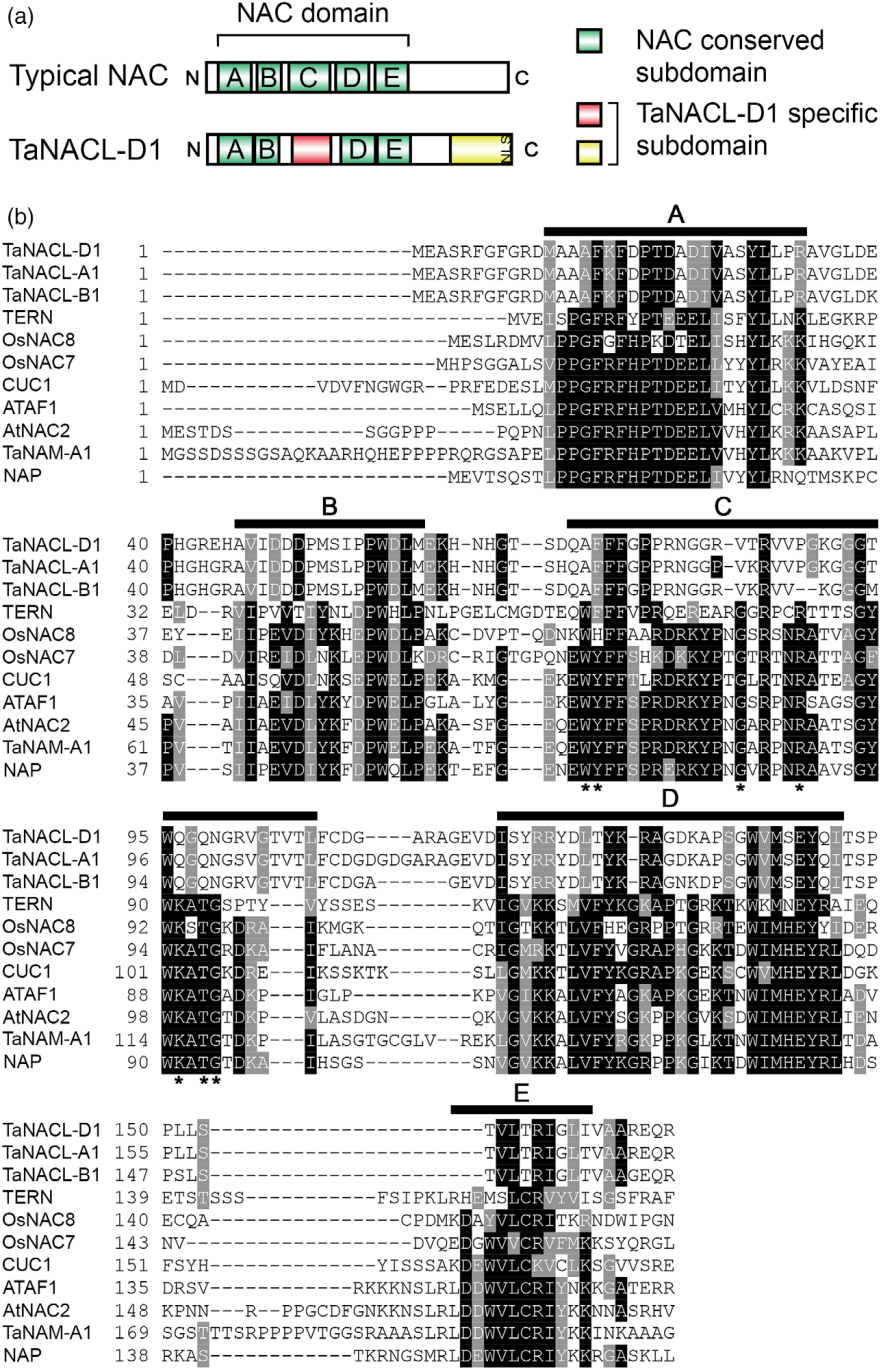
TaNACL‐D1 protein sequence features. (a) Graphical representation of TaNACL‐D1 domains and organization. (b) Sequence alignment of NAC domains. TaNACL‐D1, homeologs and other NAC transcription factors representing different phylogenetic subgroups are aligned. Conserved NAC subdomains (A‐E) are underlined. Identical and similar residues are shaded in black and grey, respectively. Asterisk symbol indicates the conserved residues within motif C that are divergent for TaNACL‐D1. Abbreviations: NLS, nuclear localization signal; TERN, tobacco elicitor‐responsive; OsNAC8, *Oryza sativa* NAC8; OsNAC7, *Oryza sativa* NAC7; CUC1, cup‐shaped cotyledon1; ATAF1, *Arabidopsis* transcription activation factor 1; AtNAC2, *Arabidopsis thaliana* NAC2; TaNAM‐A1, *Triticum aestivum* no apical meristem‐A1; NAP, NAC‐like, activated by AP3/PI.

The TaNACL‐D1 protein sequence encodes a NAC domain (IPR003441) in the N‐terminal region (residues 12–163; Figure 1a,b). A typical NAC domain consists of a series of 5 conserved subdomains, referred to as the A, B, C, D and E subdomains (Figure 1a,b) (Ooka *et al*., 2003; Pereira‐Santana *et al*., 2015). TaNACL‐D1 and its homeologous protein sequences (TaNACL‐A1 and TaNACL‐B1) were aligned with typical NAC transcription factors representing different NAC phylogenetic subgroups (Ooka *et al*., 2003). Protein sequences alignment indicates that TaNACL‐D1 and homeologs are not conserved in the C subdomain, as key residues within the subdomain are divergent (Figure 1b). This finding was confirmed using the motif analysis program MEME (http://meme-suite.org/), which uncovered the A, B, D and E subdomains in TaNACL‐D1 and its homeologs, but not the C subdomain (Figure S1a,b). In addition, MEME analysis highlighted that TaNACL‐D1 possesses an alternative putative subdomain located between the B and D NAC subdomains (Figures 1a,b and S1a,b). MEME analysis also revealed a region specific to TaNACL‐D1 and its homeologs at the C‐terminal (Figure 1a and motif 7 in Figure S1a,b). Recently, a study by Borrill *et al*. (2017) of the wheat NAC transcription family also identified a novel C‐terminal motif conserved among some NAC proteins from the phylogenetic subgroup h in which TaNACL‐D1 was found (Borrill *et al*., 2017). Interestingly, this subgroup h specific motif is similar to a section of the C‐terminal region predicted within TaNACL‐D1 and includes a continuous stretch of positively charged lysines (K) or arginines (R) coding for a putative monopartite Nuclear Localization Signal (NLS) identified using NLS mapper (Kosugi *et al*., 2009b) (Figures 1a and S1b, S2).

TaNACL‐D1 orthologues were found only within *Poaceae*, varying from 89 to 37% protein identity (for *Aegilops tauschii* and *Setaria italica,* respectively). Protein sequence alignment of TaNACL‐D1 orthologues and homeologs indicated that the *Triticeae* tribe (*Aegilops tauschii*,* T. aestivum, Triticum monococcum* and *Triticum urartu*) share high similarity in both the N‐ to the C‐terminal regions of the protein; of particular note is the similarity in both the NAC domain and the C‐terminal region wherein the predicted NLS is conserved. For the other *Poaceae* (*Brachypodium distachyon*,* Hordeum vulgare*,* Oryza sativa Indica*,* Setaria italica* and *Sorghum bicolor*) the C‐terminal part is very dissimilar and, furthermore, the NLS is absent (Figure S2). Thus, we conclude that TaNACL‐D1 is a *Poaceae* divergent NAC transcription factor that encodes NAC C‐terminal region specific to the *Triticeae*.

### TaNACL‐D1 is a transcription factor

**Figure 2:**
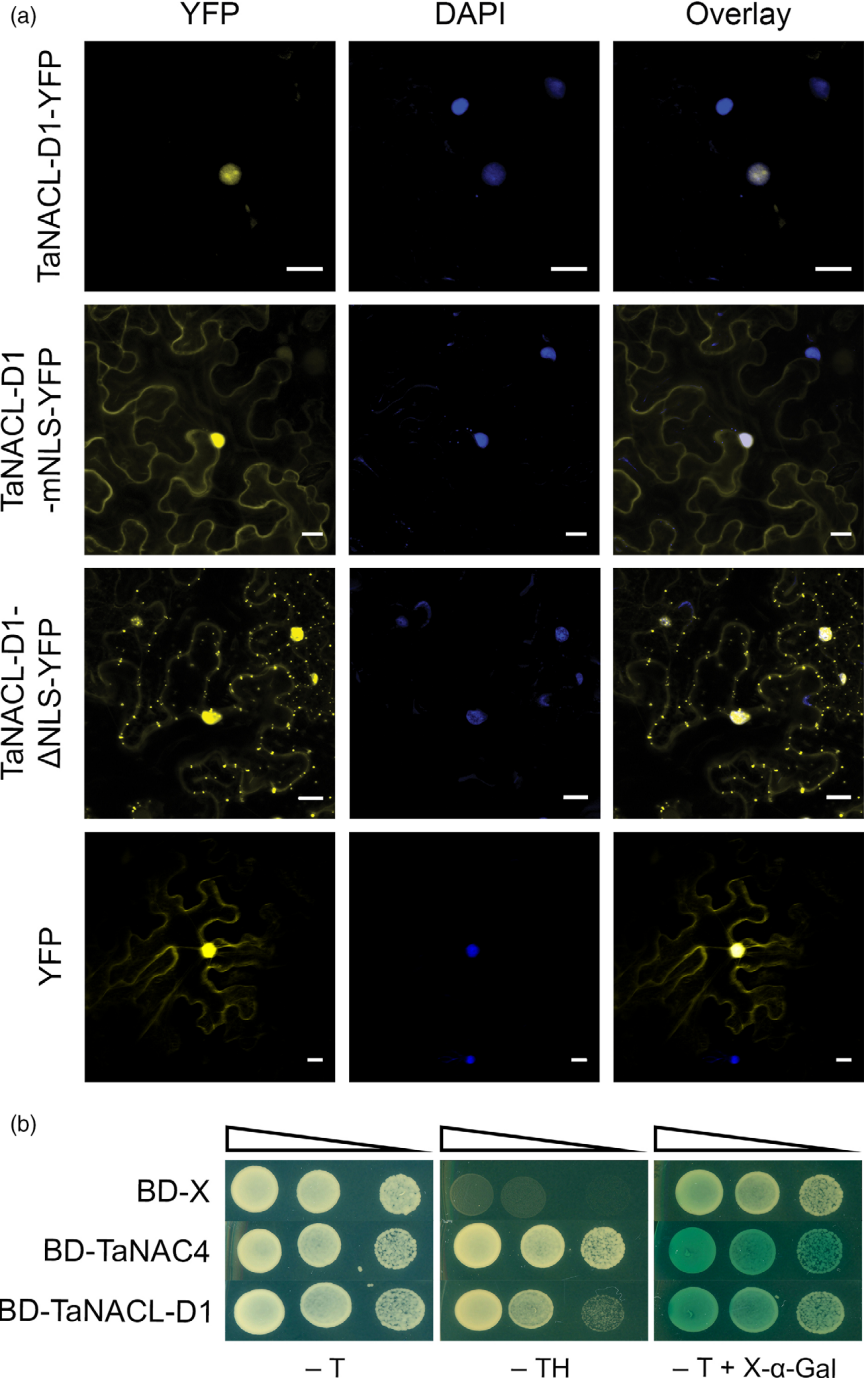
Subcellular localization and transactivation activity of TaNACL‐D1. (a) Microscopic analysis of TaNACL‐D1 within the leaf epidermal cells of tobacco. *Nicotiana benthamiana* leaves were transiently transformed with *Agrobacterium tumefaciens* harboring either the vector TaNACL‐D1‐YFP (TaNACL‐D1 fused to the yellow fluorescent protein), TaNACL‐D1‐mNLS‐YFP mutated in the Nuclear Localization Signal (NLS) or TaNACL‐D1‐ΔNLS‐YFP truncated in the NLS. Cells were observed by confocal microscopy. YFP or 4′,6‐diamidino‐2‐phenylindole (DAPI) images are shown both separately and as an overlay. Scale bar indicates 20 μm. (b) Transactivation activity of TaNACL‐D1 in yeast. The plasmids containing the genes TaNAC4 (positive control) or TaNACL‐D1 and the control plasmid pGBKT7 (BD‐X: empty vector) were expressed in Y2HGold yeast strain. The yeast transformants were incubated for 3 d at 28 °C under selective Trp/His drop‐out medium (‐TH) or non‐selective Trp drop‐out medium (‐T). α‐galactosidase activity encoded by α‐galactosidase (*MEL1*) was examined with the chromogenic substrate X‐α‐gal included in the medium. Serial dilutions (1/10) of the yeast transformants are shown by narrowing triangle.

As described, the C‐terminal region of TaNACL‐D1 encodes a predicted NLS (DKSRVKRKRRRYG), suggesting that TaNACL‐D1 can be localized in the nucleus. To investigate the TaNACL‐D1 subcellular localization, TaNACL‐D1 was fused to the yellow fluorescent protein (YFP) (TaNACL‐D1‐YFP) and the fusion was transiently expressed in *Nicotiana benthamania* leaves via *Agrobacterium tumefaciens* infiltration. Confocal microscopy revealed that TaNACL‐D1‐YFP was restricted within the DAPI‐stained nuclei (Figure 2a), whereas both YFP and fusion proteins in which the NLS was either truncated (TaNACL‐D1‐ΔNLS‐YFP) or mutated (DKSRVIPIPGPYG; TaNACL‐D1‐mNLS‐YFP) were detected in both the nucleus and the cytoplasm (Figure 2a). The expression of the different YFP fusions was confirmed via western blot analysis (Figure S3a). Thus, we demonstrated that the C‐terminal NLS sequence is important for restricting TaNACL‐D1 to the nucleus.

A yeast transactivation assay was used to test if TaNACL‐D1 can regulate gene transcription. TaNAC4, a previously wheat characterized NAC transcriptional activator, was used as a positive control (Xia *et al*., 2010). The GAL4 DNA binding domain (BD) was fused to either TaNACL‐D1 or TaNAC4 generating the constructs BD‐TaNACL‐D1 and BD‐TaNAC4. Yeast transformed with either fusion construct or with the empty vector (BD‐X) were able to grow on medium lacking tryptophan (‐T), whereas, only yeast cells with BD‐TaNACL‐D1 and BD‐TaNAC4 grew on medium lacking both tryptophan and histidine (‐TH) or turned blue in the presence of the substrate X‐α‐GAL in the medium (−T + X‐α‐GAL) (Figure 2b). These results indicate that BD‐TaNACL‐D1 and BD‐TaNAC4 can activate both reporter genes *HIS3* and *MEL1*. Protein expression from constructs was confirmed by western blot analysis (Figure S3b). Thus, we demonstrated that TaNACL‐D1 is a transcriptional activator.

### TaNACL‐D1 transcription factor interacts with TaFROG

**Figure 3:**
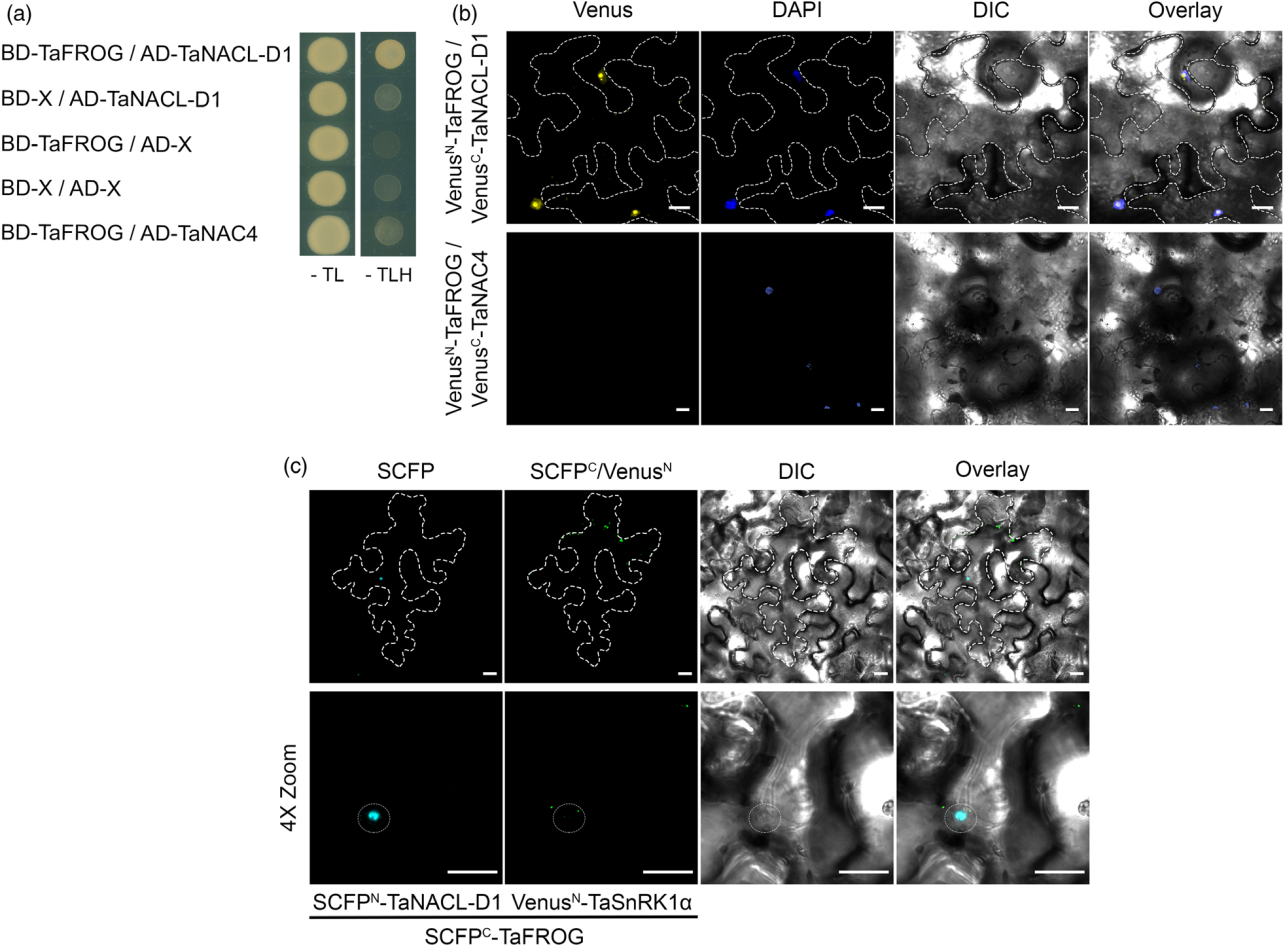
Interaction of TaFROG with TaNACL‐D1. (a) Yeast two‐hybrid assay using the yeast co‐transformed with TaNACL‐D1 and TaFROG cloned in the Gal4 bait/prey vectors. Yeast was grown for seven d under selective Trp/Leu/His drop‐out medium (‐TLH) or non‐selective Trp/Leu drop‐out medium (‐TL) conditions. (b‐c) *In planta* protein‐protein interaction visualized by the bimolecular fluorescence complementation (BiFC) assay for TaFROG/TaNACL‐D1 complex (b) or by the multicolor bimolecular complementation (mcBiFC) assay for TaFROG/TaNACL‐D1 and TaFROG/TaSnRK1α complex in the same cell (c). Confocal microscopy images of representative *Nicotiana benthamiana* epidermal leaf cells expressing proteins fused to N‐ or C‐terminal part of the Venus fluorescent protein or fused to N‐ or C‐terminal part of the SCFP (super cyan fluorescent protein) as indicated. Venus, DAPI (4′,6‐diamidino‐2‐phenylindole) (b) or SCFP, chimeric SCFP^C^/Venus^N^ (c) fluorescence and Differential Interference Contrast (DIC) images are shown both separated and as an overlay. In (b) and (c) margin of the cells expressing BiFC signal are outlined with broken lines. In (c) 4X zoom images, nucleus is outlined with a white dotted line. Scale bar indicates 20 μm. In (a) and (b), TaNAC4 was used as a negative control.

TaNACL‐D1 was originally identified using TaFROG as bait in a yeast two‐hybrid screen of a wheat cDNA library (Perochon *et al*., 2015). We used a GAL4‐based yeast two‐hybrid system to validate this interaction. Yeast transformed with a construct carrying the GAL4 DNA binding domain fused to TaFROG (BD‐TaFROG) and another carrying the GAL4 activation domain fused to TaNACL‐D1 (AD‐TaNACL‐D1), were able to grow on selective medium lacking tryptophan, leucine and, histidine (‐TLH) (Figure 3a). On the contrary, no growth was observed when we combined constructs carrying TaFROG and either another wheat NAC (TaNAC4) or the empty vector (Figure 3a). The expression of the different fusion proteins was verified by western‐blot analysis (an exception being that AD‐TaNAC4 protein could not be detected; (Figure S3c,d). Thus, we confirmed that TaNACL‐D1 interacts with TaFROG in yeast cells.

A Bimolecular Fluorescent Complementation (BiFC) system (Gehl *et al*., 2009) was used in order to assess if the interaction between TaNACL‐D1 and TaFROG can occur *in planta*. TaNACL‐D1 and TaFROG were fused at their N‐terminal with either the N‐ or C‐terminal part of Venus, which is an improved version of YFP. Resulting protein fusions were transiently expressed in *N. benthamiana* leaves and analyzed using confocal microscopy. When Venus^N^‐TaFROG and Venus^C^‐TaNACL‐D1 were combined, Venus signal was restricted to the DAPI‐stained cell nucleus (Figure 3b). The nuclear localized TaNAC4 was used as a negative control (Xia *et al*., 2010) and the combination Venus^N^‐TaFROG and Venus^C^‐TaNAC4 gave no YFP signal (Figure 3b). Thus, we demonstrated that TaNACL‐D1 can interact with TaFROG *in planta,* specifically in the nucleus.

### TaFROG forms distinct protein complexes with TaNACL‐D1 and TaSnRK1α

We previously identified TaSnRK1α as a TaFROG‐interacting protein (Perochon *et al*., 2015). Therefore, as both TaNACL‐D1 and TaSnRK1α can interact with TaFROG, we tested whether TaNACL‐D1 can interact with TaSnRK1α using the same BiFC system. As reported in Perochon *et al*. (2015) we observed Venus signal in cytosolic bodies resulting from the interaction between TaFROG and TaSnRK1α with the combination Venus^N^‐TaFROG and Venus^C^‐TaSnRK1α, but no Venus signal was observed with the combination Venus^N^‐TaNACL‐D1 and Venus^C^‐TaSnRK1α (Figure S4). Thus, we conclude that TaNACL‐D1 doesn't interact with TaSnRK1α *in planta*. TaFROG is an IDP, some of which have been shown to function as hubs in signaling networks (Wright and Dyson, 2015). To test if TaFROG might be a hub between TaNACL‐D1 and TaSnRK1α, we co‐expressed these three proteins using the multicolor BiFC (mcBiFC) system that enables within‐cell visualisation of protein‐protein complexes wherein a common protein interacts with two different partners (Waadt *et al*., 2008). With the mcBiFC system, the interaction of tagged proteins results in the reassociation of the cyan fluorescent protein SCFP3A (SCFP^N^/SCFP^C^), Venus (Venus^C^/Venus^N^) and the chimeric green fluorescent protein SCFP^C^/Venus^N^, and all three of these exhibit distinct emission spectra (Waadt *et al*., 2008). Therefore TaNACL‐D1 and TaFROG were fused at their N‐terminal with either the N‐ or C‐terminal part of SCFP. Resulting protein fusions and Venus^N^‐TaSnRK1α were transiently expressed in *N. benthamiana* leaves and interactions were analyzed. Vaidating the results observed for Venus BiFC, coexpression of SCFP^N^‐TaNACL‐D1 and SCFP^C^‐TaFROG resulted in SCFP signal in plant cell nucleus due to the interaction between TaFROG with TaNACL‐D1 (Figure 3c). Moreover, simultaneously in the same cell, we found SCFP^C^/Venus^N^ fluorescent signal in cytosolic bodies due to the interaction of TaFROG and TaSnRK1α (Figure 3c). Visualization of both TaFROG/TaNACL‐D1 and TaFROG/TaSnRK1α interaction pairs within the same cell in a distinct subcellular localization suggest that TaFROG is not a hub between TaNACL‐D1 and TaSnRK1α. Protein expression from all the BiFC and mcBiFC constructs was confirmed by western‐blot analysis (Figure S3e–h). Taken together, these experiments demonstrated that TaFROG could simultaneously form distinct protein complexes with TaNACL‐D1 and TaSnRK1α *in planta*.

### TaNACL‐D1 is responsive to *Fusarium graminearum* and its mycotoxin DON

**Figure 4:**
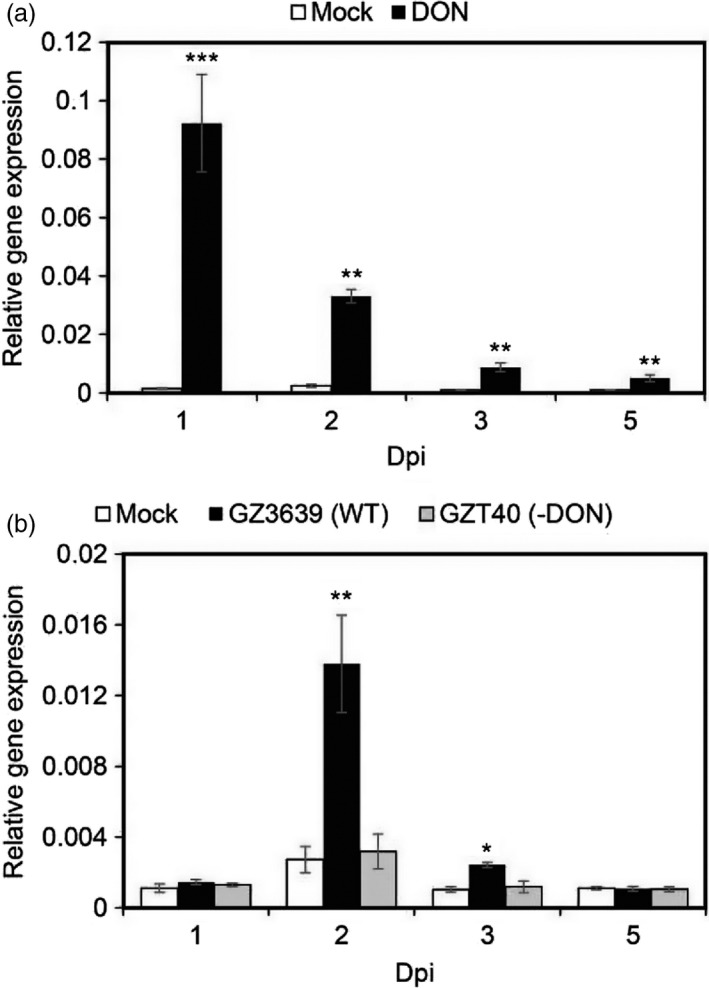
*TaNACL‐D1* transcript levels in wheat heads after treatment with DON or *Fusarium graminearum*. *TaNACL‐D1* gene expression in wheat heads treated with DON (a) or *F. graminearum* (b) was assessed via qRT‐PCR. *TaPP2AA3* and *TaYLS8* housekeeping genes were used as internal reference to calculate the relative expression of *TaNACL‐D1* using the formula 2^−(Ct target gene−Ct average housekeeping genes)^. Wheat (*Triticum aestivum*) spikelets were treated with either DON, wild type *F. graminearum* strain GZ3639, its DON‐minus mutant derivative GZT40 or mock. The tissues were harvested at various days post‐inoculation (dpi) as indicated. Results represent the mean of three (a) or two (b) trials and in each trial 4 heads received each treatment combination and error bars indicate ± SEM (a, *n *= 12; b, *n *= 8). Asterisks show significant differences between treatments and mock (Kruskal‐Wallis test; *, *P *<* *0.05; **, *P *<* *0.01; ***, *P *<* *0.001).

To form a complex in plants, interacting proteins need to be co‐expressed in the same subcellular compartment, tissue and under similar environmental conditions. *TaFROG* was previously shown to be expressed in wheat tissue almost exclusively in response to *F. graminearum* and the *Fusarium* mycotoxin DON (Perochon *et al*., 2015). DON is also a virulence factor that aids the fungus colonize plant tissue (Bai *et al*., 2002; Proctor *et al*., 1995). Gene homeolog‐specific real‐time quantitative reverse transcriptase PCR (qRT‐PCR) analysis was used to determine if *TaNACL‐D1* was expressed under the same conditions as *TaFROG*. Comparable to *TaFROG*, the basal expression of *TaNACL‐D1* was very low in mock‐treated as compared to DON‐treated heads. *TaNACL‐D1* was DON‐induced at every time point tested and peaked at 1 day post‐inoculation (dpi) (Figure 4a). Based on this expression pattern, we hypothesized that like *TaFROG*,* TaNACL‐D1* might be induced by *F. graminearum* in a toxin‐dependent manner. Indeed, *TaNACL‐D1* was activated by wild type *F. graminearum* strain GZ3639 and its expression peaked at 2 dpi, but it was not activated in response to GZT40, which is a DON‐minus mutant derivative of this *F. graminearum* strain (Figure 4b). Interestingly, *TaFROG* was reported to have exactly the same pattern of expression, with a peak of expression at 2 dpi associated with DON‐production by *F. graminearum* (Perochon *et al*., 2015).

There are several publicly available RNAseq studies for wheat development/tissues and FHB experiments (Kugler *et al*., 2013; Pfeifer *et al*., 2014; Pingault *et al*., 2015). Analyzing these data sets within Expression Atlas (Petryszak *et al*., 2016), we confirmed that *TaNACL‐D1, TaFROG* and their homeologs all presented the same temporal expression profile in response to FHB. In two independent experiments extracted from Expression Atlas and corresponding to the experiments E‐MTAB‐1729 (Kugler *et al*., 2013) and E‐MTAB‐4222, all *TaNACL‐D1* and *TaFROG* variants were transcribed at 30 hours post‐inoculation with a peak at 48–50 hours, but were not detected at very early time points (3–24 h). This activation was not dependent on the presence or absence of the resistance QTL, *Fhb1* and *Qfhs.ifa‐5A* (Figure S5a,b). Additionally, we deduced that the basal expression of *TaNACL‐D1, TaFROG* and their homeologs in healthy tissue was minimal in different wheat organs and at different developmental stages, with maximum transcript levels occurring in wheat spike and grain (Figure S5c,d). Thus, we demonstrated that *TaNACL‐D1* and *TaFROG* are co‐expressed in wheat heads in response to DON and *F. graminearum*.

### *TaNACL‐D1* enhances FHB resistance

**Figure 5:**
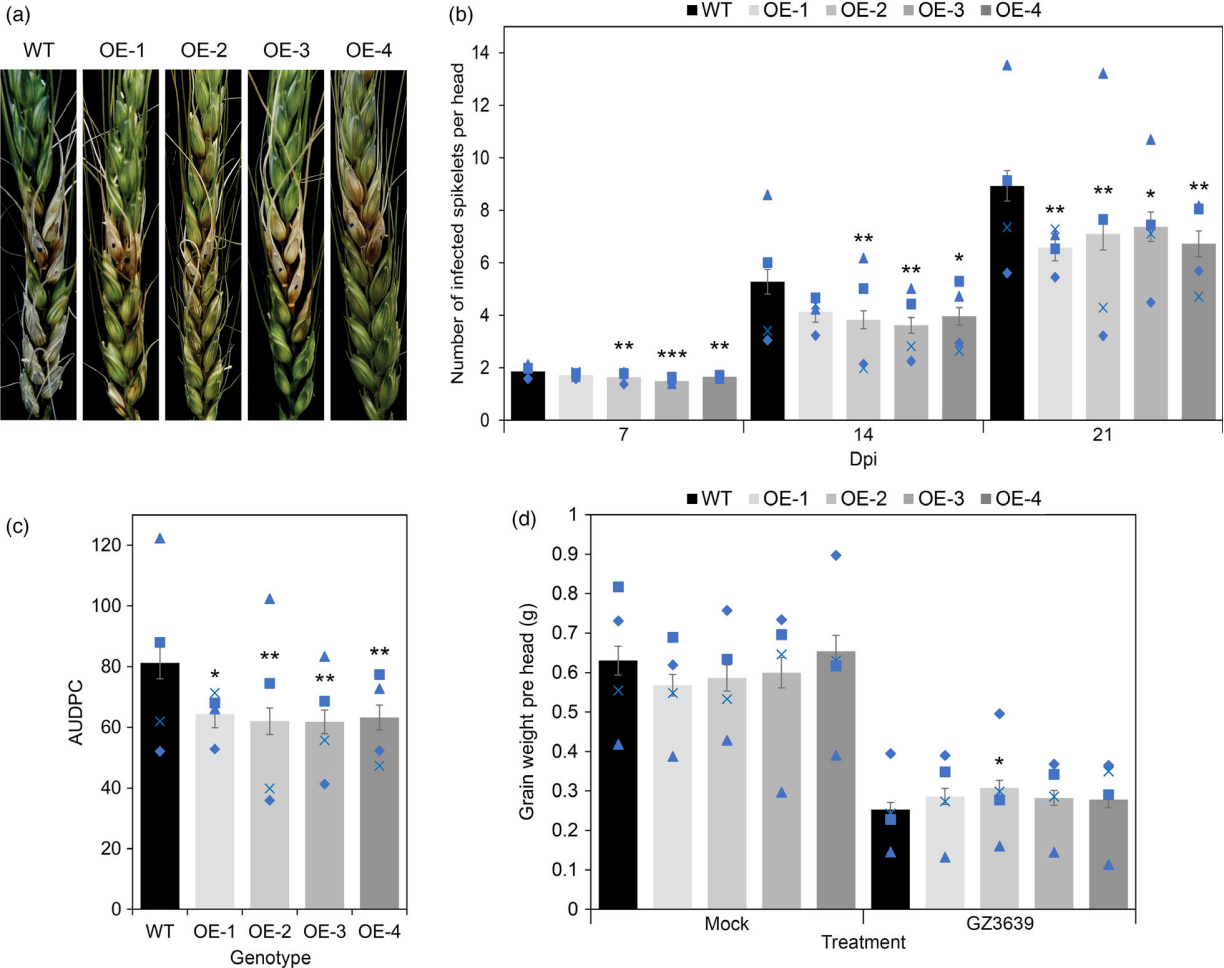
Effect of *TaNACL‐D1* overexpression on wheat (*Triticum aestivum*) Fusarium head blight resistance. At mid‐anthesis, central flowering spikelets from control plants (WT) or overexpressing lines (OE‐1, OE‐2, OE‐3 and OE‐4) were point‐inoculated with *Fusarium graminearum* strain GZ3639. Disease was assessed at different days post‐inoculation (dpi) and data presented correspond to the (a) symptoms of FHB on the *TaNACL‐D1* overexpression lines at 21 d after point inoculation, (b) to the score of infected spikelets per head at 7, 14 and 21 days, (c) to the area under the disease progress curve (AUDPC). (d) Grain yield in wheat heads mock‐inoculated (mock) or *F. graminearum*‐inoculated (GZ3639). Results represent the mean of four trials (within each, 15–25 heads per genotype were subjected to each treatment) and error bars indicate ± SEM (a, b: *n *= 80–84). Each blue shape represents the average value of one of the four individual trials. Asterisks show significant differences compared to the WT (Mann‐Whitney *U* test; *, *P *<* *0.05; **, *P *<* *0.01; ***, *P *<* *0.001).

In a previous study, we demonstrated that TaFROG enhances resistances to FHB (Perochon *et al*., 2015). Therefore, we hypothesize that its interactor TaNACL‐D1 might have a role in FHB resistance in wheat. To test this hypothesis, we generated 31 transgenic lines overexpressing *TaNACL‐D1* in wheat (cv Fielder) under the control of the rice actin promoter. Four independent homozygous lines were generated: OE‐1, OE‐2, OE‐3 and OE‐4. The T‐DNA copy number and the presence of the transgene integration was confirmed by PCR and gene overexpression was confirmed by qRT‐PCR (Figure S6a,b). Transgenic lines OE1, OE‐2, OE‐3 and OE‐4 exhibited a 143, 40, 50 and 123‐fold increase, respectively, in *TaNACL‐D1* expression compared to wild type plants. We evaluated the effect of *TaNACL‐D1* overexpression on the spread of FHB symptoms after point inoculation with the pathogen. Results showed that wild type cv Fielder had an average of 1.9, 5.3 and 8.9 diseased spikelets at 7, 14 and 21 days, respectively, post‐treatment. All transgenic lines exhibited less disease symptoms with significant reductions at 21 dpi and for all the time points for OE‐2, OE‐3 and OE‐4 (Figure 5a). Reductions of 26, 20, 17 and 25% were observed at 21 dpi for OE‐1, OE‐2, OE‐3 and OE‐4, respectively, relative to wild type plants. Furthermore, the disease progression (AUDPC), calculated using disease scores from 7, 14 and 21 dpi, was significantly lower (21–24%) for all the transgenic lines compare to wild type plants (Figure 5b).

Grain yield is an important agronomic trait negatively affected by FHB. Therefore, at harvest we measured the number of grain per head and the average individual grain weight (Figure S7) and used this data to calculate the grain yield, expressed as the average grain weight per treated head (Figure 5c). The grain yield in all the transgenic lines were comparable to the wild type in non‐fungal treated plants (mock, Figure 5c), indicating that *TaNACL‐D1* overexpression has no negative or positive effect on grain yield in healthy plants. Yield loss due to FHB was slightly less for OE‐1, OE‐2, OE‐3 and OE‐4 with 50, 47, 53, 57% yield reduction, respectively, compared to 60% for wild type spikelets. However only OE‐2 had a significant higher grain yield than the wild type in *Fusarium*‐treated heads (Figure 5c).

DON resistance is a component of FHB resistance (Gunupuru *et al*., 2017). We hypothesized that like TaFROG, TaNACL‐D1 might enhance both FHB and DON resistance (Perochon *et al*., 2015). Therefore, transgenic lines and wild type plants were point inoculated with DON and DON‐damaged spikelets were observed at different time points post‐toxin treatment. The trend was for reduced DON‐induced damage in transgenic lines compared to wild type plants, but reductions were not statistically significant (Figure S8). Thus, we conclude that the overexpression of *TaNACL‐D1* provided quantitative resistance to FHB but cannot conclude that this toxin‐responsive gene significantly affects DON resistance, at least under the conditions analyzed herein.

## Discussion

This study characterized a *Poaceae‐*divergent NAC‐like transcription factor that interacts with the *Pooideae‐*specific orphan protein TaFROG and enhances wheat's resistance to FHB disease. Given the importance of FHB disease, both economically and toxicologically (i.e. mycotoxin contamination of grain), it is gratifying to add another gene to the relatively short list with potential to contribute to FHB resistance. Other such genes in wheat include pore‐forming toxin‐like gene (*PFT*), the *TaWRKY70* transcription factor, the defensin protein *α‐1‐purothionin, β‐1,3‐glucanase, TaFROG*, and the key regulator of the salicylic defense signaling pathway, non‐expressor of pathogenesis related (*NPR1*) (Kage *et al*., 2017; Mackintosh *et al*., 2007; Makandar *et al*., 2006; Perochon *et al*., 2015; Rawat *et al*., 2016). *PFT* and *TaWRKY70* are within mapped FHB resistance QTL *Fhb1* and *QTL‐2DL,* respectively (Kage *et al*., 2017; Rawat *et al*., 2016). Previous studies have mapped FHB resistance QTL to group 5 chromosomes (Bourdoncle and Ohm, 2003; Jia *et al*., 2005; Klahr *et al*., 2007; Paillard *et al*., 2004; Yang *et al*., 2005). While neither *TaNACL‐D1* nor its *5B* homeolog map to any of these QTL, *TaNACL‐A1* maps within the QTL *Qfhs.ifa‐5A* interval (but it should be noted that this QTL represents a large interval) (Buerstmayr *et al*., 2002, 2003; Xue *et al*., 2011b).

In general, homeologous *NAC* genes share similar expression patterns (Borrill *et al*., 2017). In our *in silico* analysis, *TaNACL‐D1* and homeologs all have low basal expression and are induced by *F. graminearum* to a comparable extent. The responsiveness of *TaNACL‐D1* to both *F. graminearum* and DON led us to speculate that the FHB resistance conferred by this gene is underpinned by enhanced resistance to the virulence factor DON (as is the case for *TaFROG;* Perochon *et al*., 2015). But reductions in DON‐induced bleaching due to overexpression of *TaNACL‐D1* were not statistically significant. It may be that the cv Fielder used for gene overexpression studies has some basal level of DON resistance that masks the effects of *TaNACL‐D1* on DON sensitivity. This hypothesis is based on a comparison of the relatively low DON‐induced bleaching levels for cv Fielder observed herein and in Perochon *et al*. (2015) with the higher levels previously observed for the DON‐susceptible cv Remus (Ansari *et al*., 2007; Lemmens *et al*., 2005).

Though, there is no statistical evidence in this study showing a correlation between FHB resistance due to *TaNACL‐D1* overexpression and grain development, there was a trend for reduced FHB‐associated yield loss due to the overexpression of *TaNACL‐D1*. Silencing of DON/FHB resistance genes encoding a wheat ABC transporter *TaABCC3.1* (Walter *et al*., 2015) and a leucine rich receptor kinase (*TaLRRK‐6D*) did not significantly enhance yield loss due to toxin/disease under glasshouse conditions (Walter *et al*., 2015; Thapa *et al*., 2018). Field trials using spray inoculation of *Fusarium* on whole heads are needed to determine the true impact of these genes and *TaNACL‐D1* on yield loss due to FHB. In the absence of disease, there was no evidence that *TaNACL‐D1* overexpression affected grain development. This distinguishes *TaNACL‐D1* from *TaABCC3.1* and *TaLRRK‐6D* that both positively contributed to grain development (Walter *et al*., 2015, Thapa *et al*., 2018). And a genome‐wide expression profiling study in wheat (FHB susceptible cultivar) (Chetouhi *et al*., 2016) showed that many specific *F. graminearum* responsive transcripts are involved in processes linked to grain development.

The NAC family is one of the largest plant specific transcription factor gene families (Olsen *et al*., 2005). In wheat, a genome‐wide sequence analysis of the NAC family identified 453 NACs belonging to eight main phylogenetic groups (a‐h) (Borrill *et al*., 2017). Only a few have been functionally characterized in wheat and these are associated with plant development, nitrate response and environmental stress responses (Chen *et al*., 2016; He *et al*., 2015; Uauy *et al*., 2006; Xue *et al*., 2011a). With respect to their involvement in disease responses, wheat NACs (*TaNAC1*,* TaNAC21/22, TaNAC30*) have been shown to have a negative role in regulating resistance to the stripe rust disease (Feng *et al*., 2014; Wang *et al*., 2015; Wang *et al*., 2018). To our knowledge, *TaNACL‐D1* is the only wheat NAC functionally characterized from the group h and the first plant NAC shown to play a role in FHB resistance.

TaNACL‐D1 and its homeologs differ from most NACs in that they lack one of the characteristic subdomains of NAC proteins (i.e. the C subdomain). Similarly, the switchgrass NAC subgroup XV lacks the C subdomain and Yan *et al*. (2017) proposed that this difference might contribute to the functional divergence and specification in biological processes. The C‐terminals of NACs contain the transcriptional activation/repression regions with highly divergent sequences. Despite this divergence, common C‐terminal motifs are usually predicted but their functional significance remains poorly understood (Borrill *et al*., 2017; Olsen *et al*., 2005; Ooka *et al*., 2003). Sequence and motif analysis of TaNACL‐D1, homeologs and orthologues revealed a putative NAC C‐terminal region specific to the *Triticeae* that might participate in a subfamily‐specific functionality. Within this region we identified a NLS motif crucial to restrict the nuclear localization of TaNACL‐D1. Interestingly, this motif corresponds to the NAC h subgroup specific motif identified in the analysis of Borrill *et al*. (2017).

We demonstrated in this study that the orphan protein TaFROG interacts with TaNACL‐D1. Another example of a plant orphan protein interacting with a transcription factor has been previously reported in *Arabidopsis:* the orphan protein Qua‐Quine Starch (QQS) binds the conserved eukaryotic transcription factor AtNF‐YC4 (*Arabidopsis* nuclear factor Y, subunit C4) (Li *et al*., 2015). Both QQS and AtNF‐YC4 modulate carbon and nitrogen allocation, but the molecular mechanism by which the complex functions is still undetermined. Recently it was shown that overexpression of *AtQQS* and *NF‐YC4* in Arabidopsis and soybean enhanced resistance/reduces susceptibility to viruses, bacteria, fungi, aphids and soybean cyst nematodes (Qi *et al.,* 2019).

We previously identified the protein kinase TaSnRK1α as a TaFROG‐interacting protein (Perochon *et al*., 2015). Thus, the identification of TaNACL‐D1 increases the repertoire of validated TaFROG‐interacting proteins and confirms that TaFROG can interact with different signaling proteins. Orphans can have a pleiotropic effect, controlling different pathways in response to environmental change (Li *et al*., 2014; Wang *et al*., 2011). In yeast the orphan Mating Depressing Factor1 (MDF1) interacts with functionally distinct signaling proteins, thus allowing the control of diverse pathways in response to environmental change. MDF1 interacts with both SNF1 (the orthologue of TaSNRK1α) to promote growth and with the transcription factor MATα2 to repress mating (Li *et al*., 2014) Whether or not TaFROG has a pleiotropic effect with its ability to bind different proteins to serve different pathways need to be investigated in more detail. However, because in this study we demonstrated the ability of TaFROG to interact with TaNACL‐D1 and TaSnRK1α in distinct subcellular locations, it is reasonable to speculate that TaFROG is involved in different signaling pathways.

In the current study, we present the functional characterization of *TaNACL‐D1,* a wheat transcription factor that interacts with the orphan TaFROG and contributes to FHB disease resistance in wheat. Future work will identify TaNACL‐D1 target genes, which will improve our understanding of its ability to enhance the wheat resistance against FHB. As TaNACL‐D1 is a divergent NAC, further studies on the role of putative specific domains identified in this study may give an insight into the functional divergence of the NACs. As the orphan TaFROG protein enhances resistance against FHB and has different interacting partners, it would be interesting to study if these interactions (TaFROG/TaSnRK1α, TaFROG/TaNACL‐D1) serve the same biological pathway and if their effects are additive. TaNACL‐D1 is a potential target for genetic engineering programs aiming to enhance FHB resistance in cereals. Determination of the degree of allelic diversity in *TaNACL‐D1* and its homeologs will provide insight into the potential of this gene and its homeologs as a marker for disease resistance breeding programs.

## Experimental procedures

### Plant material and growth conditions

Wheat (*T. aestivum*) cultivars (cvs) CM82036 and Fielder were used in this study. Wheat cv CM82036 is resistant to both FHB and DON (Buerstmayr *et al*., 2003; Lemmens *et al*., 2005), while cv Fielder is susceptible to FHB (Badea *et al*., 2013). The wheat cv CM82036 was used for gene expression studies, whereas cv Fielder and its *TaNACL‐D1* overexpression derivatives were used for disease assessment studies. Wheat seeds were germinated in darkness for 96 h at 21 °C in 90 mm petri dishes containing moist Whatman No. 1 filter paper (Whatman, Maidstone, UK). The germinated seedlings were transferred to 3 l pots containing John Innes compost No. 2 (Westland Horticulture, Dungannon, UK). All studies were carried under contained glasshouse conditions with a 25–18 °C with a 16 h : 8 h, light : dark photoperiod at 300 μmol/m^2^/s and 70% relative humidity.

### Fungal material and growth conditions

The wild type, DON‐producing *F. graminearum* strain (GZ3639) and its non‐producing derivative (GZT40) were used in this study (Proctor *et al*., 1995). Fungal mycelium was stored at −80 °C and, prior to use, was subcultured onto potato dextrose agar (PDA) (Difco) and plates were incubated at 25 °C for 5 days. Fungal spores were produced in Mung bean broth as previously described (Bai and Shaner, 1996). The spores were harvested, washed and adjusted to the required conidial concentration, all as previously described (Brennan *et al*., 2005).

### Wheat heads for gene expression studies

Gene expression studies were conducted using the RNA from the DON and FHB experiments described by Perochon *et al*. (2015). The DON experiment comprised a total of twelve heads per treatment combination (three trials, each containing four heads per treatment combination). The FHB experiment comprised a total of eight heads per treatment combination (two trials, each containing four heads per treatment combination). In each trial, treatments were applied to two central spikelets per head. RNA was extracted form one pooled sample per treatment (representing a pool of 4 heads from individual plants) and RNA was divided in two subsamples for gene expression studies.

### DNA, RNA extraction and cDNA synthesis

All DNA, RNA extractions and cDNA synthesis were done as described previously by Perochon *et al*. (2015).

### Cloning of *TaNACL‐D1* and bioinformatic analysis

After a yeast two‐hybrid screen using TaFROG as a bait, the prey fragments of positive clones obtained were amplified by PCR and sequenced (Perochon *et al*., 2015). Independent sequences corresponding to the gene *TaNACL‐D1* were used to generate a consensus sequences from which was deduced an open reading frame (ORF) and the coding sequence (CDS) using NCBI ORF finder (https://www.ncbi.nlm.nih.gov/orffinder/). Using primers designed to target the 5′ and 3′ untranslated region (UTR) of *TaNACL‐D1*, the full‐length gene was cloned by PCR from cDNA produced from DON‐treated heads of wheat cv CM82036 (TaNACL‐D1 ATG ‐27 for/ TaNACL‐D1 TGA + 89 rev, primers sequences in Table S1). Amplified fragments were cloned into the pGEM^®^‐T vector system (Promega, Madison, WI) and sequenced. Orthologues and homeologs were identified by BLASTn analysis of URGI IWGSC wheat genome database (https://wheat-urgi.versailles.inra.fr/Seq-Repository/BLAST). Multiple sequence alignments of NAC proteins were generated using Clustal Omega (http://www.ebi.ac.uk/Tools/msa/clustalo/) and Boxshade (http://www.ch.embnet.org/software/BOX_form.html) programs. Prediction of Nuclear Localization Signal (NLS) was performed using the cNLS mapper (http://nls-mapper.iab.keio.ac.jp/cgi-bin/NLS_Mapper_form.cgi) (Kosugi *et al*., 2009a,b).

### Generation of transgenic *TaNACL‐D1* overexpressing wheat plants

A wheat overexpression construct encoding the *TaNACL‐D1* gene driven by a rice *actin* promoter was generated using a Gateway cloning strategy. Briefly, *TaNACL‐D1* CDS was amplified by PCR (primers listed in Table S1), cloned into pDONR207 vector (Invitrogen, Carlsbad, CA) and subsequently recombined into the binary vector pSc4‐ActR1R2 (containing the gene of interest driven by a rice *actin* promoter (McElroy *et al*., 1990)). This vector also contains the *neomycin phosphotransferase II* (*nptII*) gene under the control of the Subterranean Clover Stunt Virus Sc4 promoter (Schünmann *et al*., 2003) and the *Arabidopsis thaliana FAD2* intron (Okuley *et al*., 1994) for selection on geneticin G418 antibiotic in tissue culture. The recombinant plasmid pEW266‐TaNACL‐D1 was electro‐transformed into *A. tumefaciens* strain AGL‐1 and subsequently into wheat embryos of cv Fielder as previously described for the *TaFROG* gene (Perochon *et al*., 2015). Transformants were selected as previously described (Perochon *et al*., 2015). For each T0 transformants, the T‐DNA copy number was determined using a qPCR assay (1 copy in lines OE‐1 and OE‐3, 2 copies in line OE‐4 and 3 copies in line OE‐2) as described by (Milner *et al*., 2018) and confirmed for the presence of the transgene insert by PCR amplification of a fragment of *TaNACL‐D1* and the NOS (nopaline synthase) terminator (primers listed in Table S1). Plants were grown to the T_4_ generation, and at each generation, leaf tissue samples were collected from the overexpression lines to check the presence of T‐DNA and to analyze the segregation ratio to identify homozygous lines. RNA extracted from leaf samples of 14 day old plants was used to study transgene expression by qRT‐PCR (primers listed in Table S1).

### Quantitative reverse transcriptase PCR analysis

qRT‐PCR analyses were conducted using the Stratagene Mx3000TM Real Time PCR as described by Perochon *et al*., 2015;. PCR primers (Table S1) used in this study were designed using genome specific primer (GSP) (Wang *et al*., 2016) and Primer3web (http://primer3.ut.ee/, (Untergasser *et al*., 2012). The specificity of the primers targeting the chromosome 5D variant was checked via PCR of DNA extracts from nullisomic‐tetrasomic lines of cv Chinese Spring (obtained from Germplasm Resources Unit, JIC, Norwich http://www.jic.ac.uk/germplasm/). *Yellow‐leaf specific gene 8* (*YLS8*,* TraesCS1D02G332500*) and *T. aestivum Protein phosphatase 2A subunit A3* (*TaPP2AA3*,* TraesCS5B02G165200*) genes were used as housekeeping genes in this study. These genes were verified not to be differentially expressed in our experiments or in publicly available RNAseq studies for FHB experiments. The threshold cycle (Ct) values obtained by qRT‐PCR were used to calculate the relative gene expression using the Equation 2^−(Ct target gene – Ct housekeeping gene)^ as described previously (Livak and Schmittgen, 2001).

### FHB assessment and DON studies

Wheat cv Fielder and its T_4_ T‐DNA homozygous transgenic derivatives (overexpressing *TaNACL‐D1*) were used for DON and FHB trials in the glasshouse under controlled environmental conditions. Wild type *F. graminearum* strain GZ3639 (Proctor *et al*., 1995) was used in FHB trials. Treatments with *Fusarium* and DON, measured levels of infection and phenotypic effect of DON were done as described previously by Perochon *et al*. (2015). This data was used to calculate disease progression, measured as the area under the disease progress curve (AUDPC) (Shaner & Finney, 1977). Heads were harvested after maturation (Zadoks growth stage 99 (Zadoks *et al*., 1974), for yield analysis. Heads were threshed and cleaned manually. Seeds were freeze‐dried for 4 days, counted and weighed using an Explorer^®^ Precision digital balance (Ohaus). Both the DON and FHB experiments were based on four trials, and in each trial a minimum of 15 heads (secondary tillers from minimum 10 plants) per genotype were subjected to each treatment.

### Yeast two‐hybrid (Y2H) analysis

Full‐length CDS of *TaNACL‐D1* and *TaNAC4* (Xia *et al*., 2010) was amplified by PCR (primers listed in Table S1), cloned into the vector pDONR207 using the Gateway cloning technology, and then recombined into bait Gateway vectors derived from pGBKT7 plasmids (Clontech, Mountain View, CA). *TaFROG* in prey vector pGAD was described in Perochon *et al*. (2015). Analysis of protein‐protein interactions was performed using the Gal4 two‐hybrid assay, as described previously (Perochon *et al*., 2010) using the Y2H Gold yeast strain (Clontech).

### Transactivation assay and X‐Gal assay

Transformed Y2H Gold yeast cells carrying the construct *TaNACL‐D1*,* TaNAC4* (positive control) cloned into pGBKT7 or empty vectors (negative control) were selected on tryptophan drop out medium (‐T) and on medium lacking both tryptophan and histidine (‐TH) to determine *HIS3* reporter gene expression. In order to test the activity of α‐galactosidase encoded by α‐galactosidase (*MEL1*) reporter gene*,* yeast transformants were incubated at 28 °C on a tryptophan drop out medium supplemented with X‐α‐Gal as described in Clontech yeast protocols handbook (Clontech).

### Subcellular localization of fluorescent proteins and bimolecular fluorescence complementation (BiFC)

The full‐length CDS of *TaNACL‐D1,* as well as variants mutated or deleted in the NLS, were cloned by PCR without the stop codon (primers listed in Table S1) into the vector pDONR207, using the Gateway cloning technology. *TaNACL‐D1* and NLS modified variants were subsequently cloned into binary vector pAM‐PAT‐P35S‐YFP (Bernoux *et al*., 2008). Resulting vectors pAM‐PAT‐P35S‐TaNACL‐D1‐YFP, pAM‐PAT‐P35S‐TaNACL‐D1‐mNLS‐YFP and pAM‐PAT‐P35S‐TaNACL‐D1‐ΔNLS‐YFP were introduced into *A. tumefaciens* strain GV3101 by electroporation. *A. tumefaciens* transformants were grown in Luria Bertani (LB) media containing 20 μg/mL gentamicin and 50 μg/mL carbenicillin and was syringe infiltrated into leaf epidermal cells of 4 week old *N. benthamiana* plants prior to microscopy observation (OD_600 nm_ = 1).

For the bimolecular fluorescence complementation (BiFC) analysis, gateway technology was used to subclone *TaFROG*,* TaNACL‐D1*,* TaNAC4 and TaSnRK1α* CDS into the pDEST‐VYNE^GW^, pDEST‐VYCE^GW^, p(MAS)DEST‐SCYCE^GW^ and pDEST‐SCYNE^GW^ (Gehl *et al*., 2009). This resulted in constructs wherein the protein was fused at the C terminal to the Venus C‐terminal fragment (Venus^C^) or SCFP3A C‐terminal fragment (SCFP^C^) or the Venus N‐terminal fragment (Venus^N^) or SCFP3A N‐terminal fragment (SCFP^N^) (Perochon *et al*., 2015). Vectors were introduced into *A. tumefaciens* strain GV3103 by electroporation. A mix of *Agrobacterium* transformants was prepared at final optical density OD_600 nm_ = 0.5 for each Venus^C^ and Venus^N^ constructs (BiFC analysis), or OD_600 nm_ = 0.4 for each Venus^N^, SCFP^N^ and SCFP^C^ constructs (multicolor BiFC analysis) plus OD_600 nm_ = 0.3 for the P19 silencing construct (http://www.plantsci.cam.ac.uk/research/davidbaulcombe/methods/protocols/pbin61-p19.doc/view). Cells were imaged for fluorescence at 1–2 days (BiFC analysis) or 3 d (TaNACL‐D1 subcellular localization and multicolor BiFC analysis) after *Agrobacterium* leaf inoculation and 4′,6‐diamidino‐2‐phenylindole (DAPI) staining as described in previous studies (Perochon *et al*., 2015). Images were captured using a confocal laser scanning microscope (Olympus fluoview FV1000) equipped with a UPLSAPO 40X objective. DAPI, SCFP, chimeric SCFP^C^/Venus^N^ and YFP excitation was performed at 405, 405, 515 and 515 nm, respectively, and emission detected in the 460–500 nm range for DAPI and SCFP, in the 520–555 nm range for the chimeric SCFP^C^/Venus^N^ and in the 530 to 630 nm range for YFP. Microscopy images were processed using FIJI software. The localization and BiFC experiments each comprised three trials, and each trial included three leaves from individual plants.

### Western blot analysis

To confirm protein expression in yeast studies (Y2H and transactivation assay), yeast strains were grown at 28 °C overnight in medium lacking the appropriate amino acid, then grown for 3–5 h in yeast peptone dextrose liquid medium. Using Y‐PER (Yeast Protein Extraction Reagent, Thermo Scientific), total protein was extracted from yeast cells following the manufacturer's instructions. To confirm protein expression in *N. benthamiana* cells (TaNACL‐D1 subcellular localization and BiFC studies), leaves were flash frozen in liquid nitrogen (N_2_) and ground with beads in a Tissuelyser II (Qiagen, Hilden, Germany). Using PEB (Protein Extraction Buffer, Agrisera), total protein was extracted from tobacco leaves, following the manufacturer's instructions. As described in Perochon *et al*. (2015), proteins were electrophoresed and transferred using a NuPAGE system (Life technologies) according to the manufacturer's instructions. Proteins transferred to nitrocellulose membrane were detected by either, an anti‐HA antibody (Roche, Basel, Switzerland) at 1/1000 dilution for the protein fused to the Venus C‐terminal fragment (Venus^C^), fused to SCFP C‐terminal (SCFP^C^) and fused to Gal4 activating domain (AD), an Anti‐c‐Myc antibody (Roche) at 1/700 dilution for the protein fused to Gal4 binding domain (BD), an Anti‐GFP antibody (Invitrogen) at 1/5000 dilution for the protein fused to the YFP or fused to the Venus N‐terminal fragment (Venus^N^), an Anti‐FLAG antibody (Sigma, Kawasaki, Japan) at 1/5000 dilution for the protein fused to SCFP N‐terminal (SCFP^N^). Following electrochemiluminescence assay, the emitted signal was imaged with the Fusion‐FX (Vilber Lourmat, Collégien, France).

### Statistical analysis

Statistical analyses were performed using the SPSS statistic version 20 software. The normality of the data distribution was evaluated with the Shapiro‐Wilk test. Gene expression, DON and FHB data sets were compared using Independent‐Samples Kruskal‐Wallis or Mann‐Whitney U tests.

## Author contributions

A.P., A.K. and F.D. designed the research; A.P. and F.D. supervised the experiments; A.P., A.K., M.V., J.J. and K.M. performed the experiments; E.W. and M.C. transformed wheat with *T. aestivum NACL‐D1* and determined the gene copy number; A.P., A.K., M.V. analyzed the data; A.P., A.K. and F.D. wrote the article with contributions from other authors. A.P. and A.K. contributed equally to this work.

## Conflict of interest

The authors have a patent pending related to this material.

## Accession numbers

TaNACL‐D1 (GB No.: MG701911) and TaNAC4 (GB No.: MG701912) were cloned from the wheat cv CM82036. ATAF1 (GB No.: OAP14514), AtNAC2 (GB No.: BAB20600), CUC1 (GB No.: BAB02571), NAP (GB No.: AEE34932), OsNAC7 (GB No.: BAA89801), OsNAC8 (GB No.: BAA89802), TaNAM‐A1 (GB No.: AIZ97664), TERN (GB No.: AB021178) were collected from the NCBI GenBank database.

## Supporting information


**Figure S1** MEME analysis of TaNACL‐D1, it's homeologs and transcription factors representing different NAC phylogenetic subgroups.
**Figure S2** Protein sequences alignment of TaNACL‐D1 homeologs and orthologues.
**Figure S3** Immunoblot analysis of the total proteins extracted from yeast and tobacco assays.
**Figure S4** Interaction of TaSnRK1α with TaFROG and TaNACL‐D1.
**Figure S5** Expression of *TaNACL‐D1* and *TaFROG* homeologs in different tissues and in response to *F. graminearum*.
**Figure S6** Molecular characterization of transgenic wheat (*Triticum aestivum*) cv Fielder overexpressing *TaNACL‐D1*.
**Figure S7** Effect of *TaNACL‐D1* overexpression on grain yield in wheat heads mock‐inoculated (mock) or *F. graminearum*‐inoculated (GZ3639).
**Figure S8** Effect of *TaNACL‐D1* overexpression on DON tolerance.

**Table S1** Primer sets used in this study.
