# Supplementary material for: A wheat NAC interacts with an orphan protein and enhances resistance to Fusarium head blight disease
Source: Plant Biotechnol J. 2019 Apr 29;17(10):1892–904. doi: 10.1111/pbi.13105 (PMC6737021; doi:10.1111/pbi.13105)
Supplement: Supplementary file 1 — Figure S1 MEME analysis of TaNACL‐D1, it's homeologs and transcription factors representing different NAC phylogenetic subgroups. Figure S2 Protein sequences alignment of TaNACL‐D1 homeologs and orthologues. Figure S3 Immunoblot analysis of the total proteins extracted from yeast and tobacco assays. Figure S4 Interaction of TaSnRK1α with TaFROG and TaNACL‐D1. Figure S5 Expression of TaNACL‐D1 and TaFROG homeologs in different tissues and in response to F. graminearum. Figure S6 Molecular characterization of transgenic wheat (Triticum aestivum) cv Fielder overexpressing TaNACL‐D1. Figure S7 Effect of TaNACL‐D1 overexpression on grain yield in wheat heads mock‐inoculated (mock) or F. graminearum‐inoculated (GZ3639). Figure S8 Effect of TaNACL‐D1 overexpression on DON tolerance. [file PBI-17-1892-s002.pdf]

Supporting Information

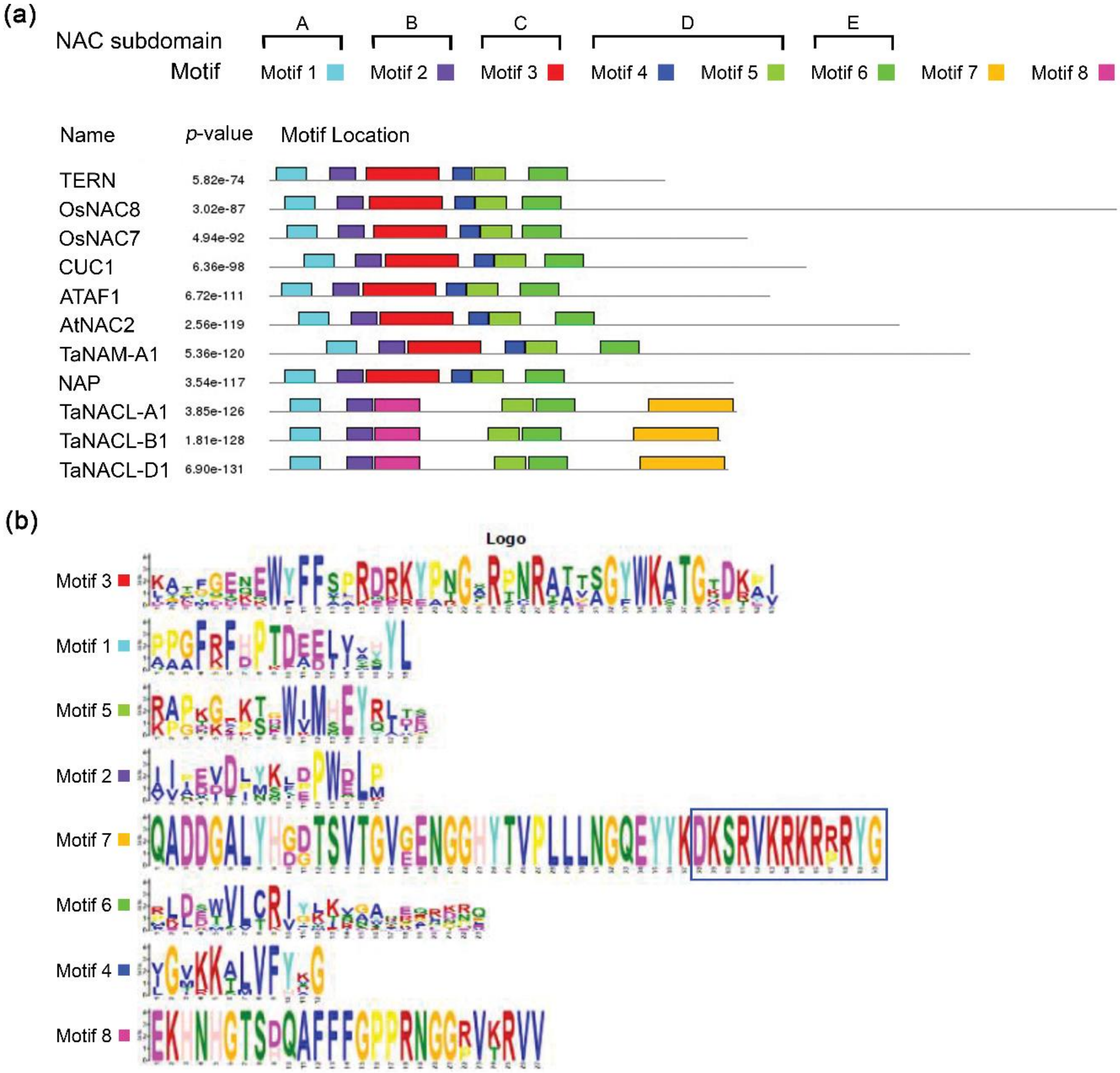

Figure S1. MEME analysis of TaNACL-D1, its homeologs and transcription factors representing different NAC phylogenetic subgroups. Multiple Em for Motif Elicitation (MEME) analysis was performed using MEME Suite Version 4.11.3 (http://meme-suite.org/tools/meme). (a) Graphical representation and location of discovered motifs. Motif 1, 2, 3 and 6 correspond to the NAC subdomain A, B, C and E, respectively. Motif 4 and 5 combined correspond to the NAC subdomain D. Motif 7 and 8 correspond to domains specific to TaNACL-D1 and its homeologs. (b) Amino acid sequences of the motifs. The blue box in motif 7 denotes the predicted monopartite NLS. Default parameters were used for the analysis: maximum number of motifs: 8; minimum motif width: 12; maximum motif width: 50; minimum sites per motif: 2; maximum sites per motif: 11. Abbreviations: TERN, tobacco elicitor-responsive; OsNAC8, *Oryza sativa* NAC8; OsNAC7, *Oryza sativa* NAC7; CUC1, cup-shaped cotyledon1; ATAF1, *Arabidopsis* transcription activation factor 1; AtNAC2, *Arabidopsis thaliana* NAC2; TaNAM-A1, *Triticum aestivum* no apical meristem-A1; NAP, NAC-like, activated by AP3/PI.

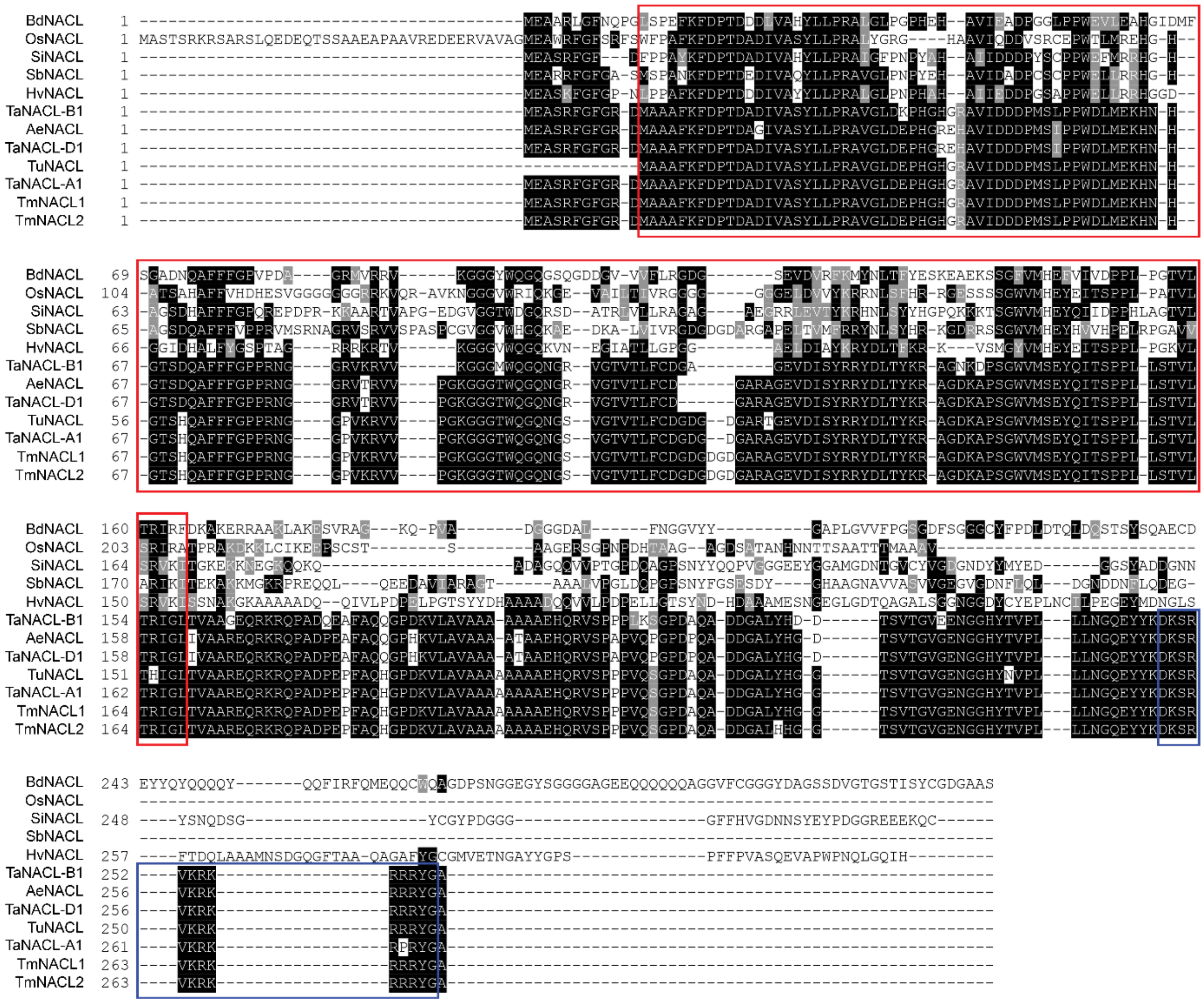

Figure S2. Protein sequences alignment of TaNACL-D1 homeologs and orthologues. TaNACL-D1 homeolog sequences were collected from the IWGSC RefSeq v1.0. Orthologues from *Aegilops tauschii* (AeNACL: EMT13761), *Brachypodium distachyon* (BdNACL: Bradi1g36200), *Hordeum vulgare* (HvNACL: MLOC_79696), *Oryza sativa Indica* (OsNACL: BGIOSGA034395), *Setaria italica* (SiNACL: Si027729m), *Sorghum bicolor* (SbNACL: SORBI_005G059900), *Triticum urartu* (TuNACL: TRIUR3_11581-T1) were collected from EnsemblPlants. Orthologues from *Triticum monococcum* (TmNACL1: TmoG3116v1_109156 and TmNACL2: TmoDV92v1_088269) were identified from the transcriptome data of (Fox *et al.*, 2014). The red and blue box denotes the NAC domains and the predicted monopartite (nuclear localisation signal) NLS, respectively. Identical and similar residues are shaded in black and grey, respectively.

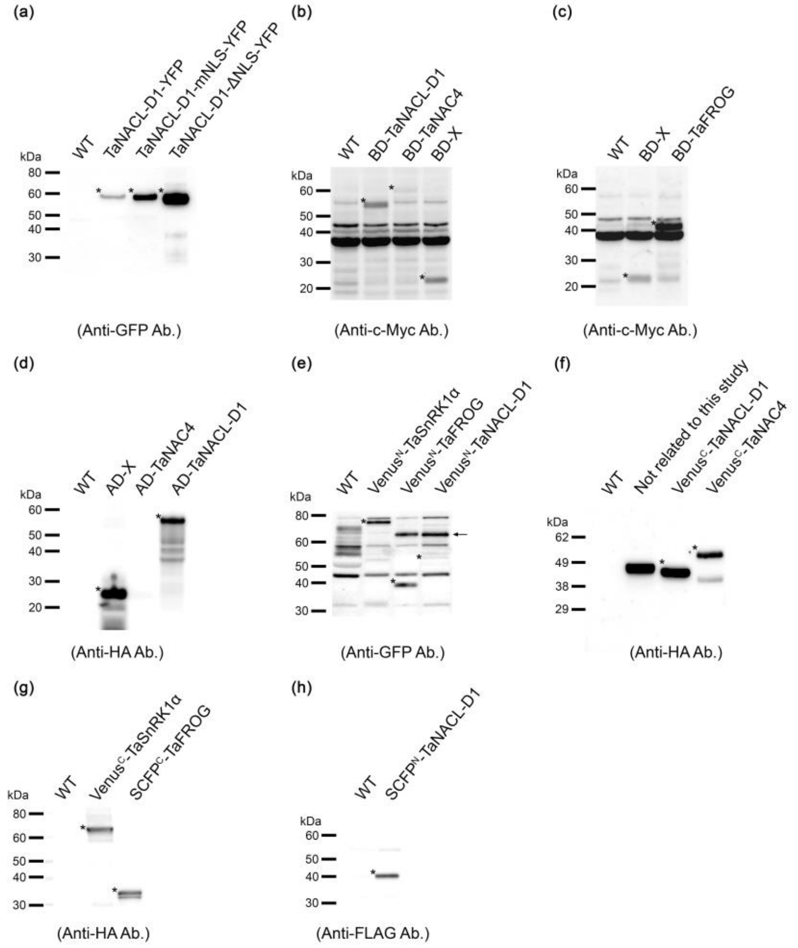

Figure S3. Immunoblot analysis of the total proteins extracted from the yeast, tobacco bimolecular fluorescence complementation (BiFC) and multicolor BiFC (mcBiFC) assays. (a) Immunoblot from TaNACL-D1 subcellular assay, (b) yeast transactivation assay, (c-d) yeast two-hybrid assay, and (e-h) BiFC and mcBiFC assays. (a) Proteins fused to the YFP (yellow fluorescent protein) were detected with an anti-GFP antibody (Ab.). (b-c) Proteins fused to the Gal4 binding domain (BD) were detected with an anti-c-Myc antibody. (d) In the case of proteins fused to the Gal4 activating domain (AD), an anti-HA antibody was used. (e-g) Proteins fused to the N- or C-terminal of the Venus protein were detected with an anti-GFP antibody or anti-HA antibody, respectively. (g-h) For proteins fused to the N- or C-terminal of SCFP, an anti-FLAG antibody or anti-HA antibody was used, respectively. The position of the different fusion proteins is indicated with an asterisk (other bands represent protein degradation products and non-specific product) or with an arrow for the detection by anti-GFP antibody of the co-expressed Venus^{C}-TaSnRK1α. The nature of the fusion protein present in each protein extract is indicated above each lane. The molecular weight are indicated on the right of the blot. The protein size expected are: (a) 57 kDa (TaNACL-D1-YFP and TaNACL-D1-mNLS-YFP), 56 kDa (TaNACL-D1-ΔNLS-YFP). (b-d) 52 kDa (BD-TaNACL-D1), 57 kDa (BD-TaNAC4), 22 kDa (BD-X), 37 kDa (BD-TaFROG), 24 kDa (AD-X), 53 kDa (AD-TaNACL-D1). (e-f) 79 kDa (Venus^{N}-TaSnRK1α), 36 kDa (Venus^{N}-TaFROG), 51 kDa (Venus^{N}-TaNACL-D1), 40 kDa (Venus^{C}-TaNACL-D1), 46 kDa (Venus^{C}-TaNAC4), 69 kDa (Venus^{C}-TaSnRK1α), 27 kDa (SCFP^{C}-TaFROG) and 41 kDa (SCFP^{N}-TaNACL-D1).

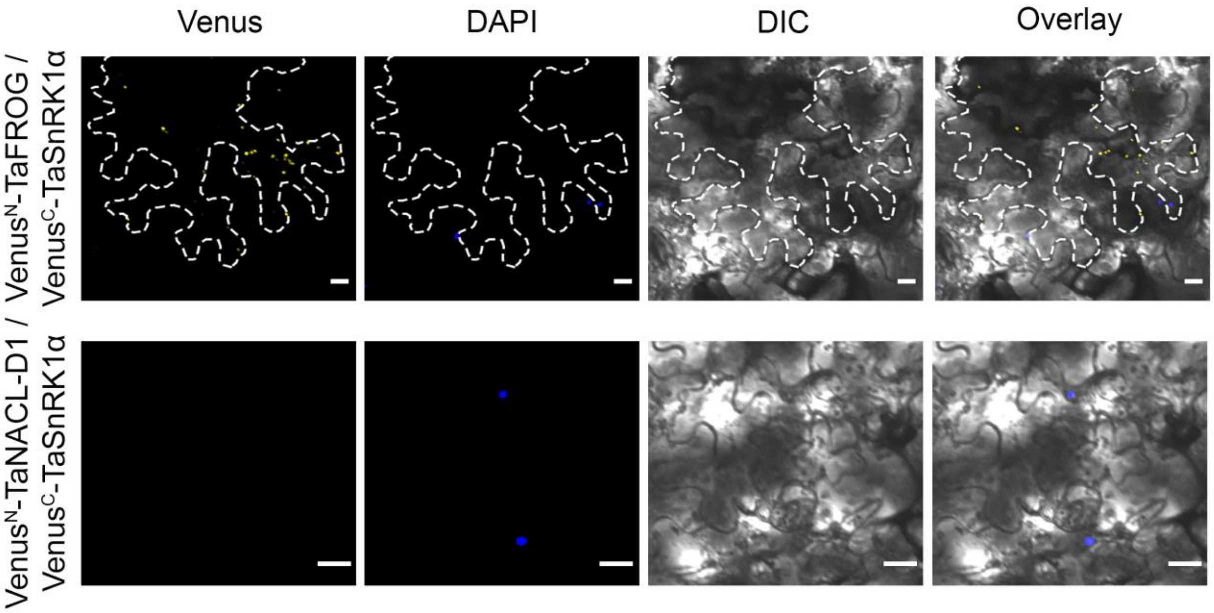

Figure S4. Interaction of TaSnRK1α with TaFROG and TaNACL-D1. *In planta* protein-protein interaction visualized by the BiFC assay. Confocal microscopy images of representative *N. benthamiana* epidermal leaf cells expressing proteins fused to N- or C-terminal part of the Venus (fluorescent protein) as indicated. Venus, DAPI (4',6-diamidino-2-phenylindole) fluorescence and Differential Interference Contrast (DIC) images are shown both separated and as an overlay. Margin of the cells expressing BiFC signal are outlined with broken line. Images were captured using a UPLANSAPO 10X objective. Scale bar indicates 20 µm.

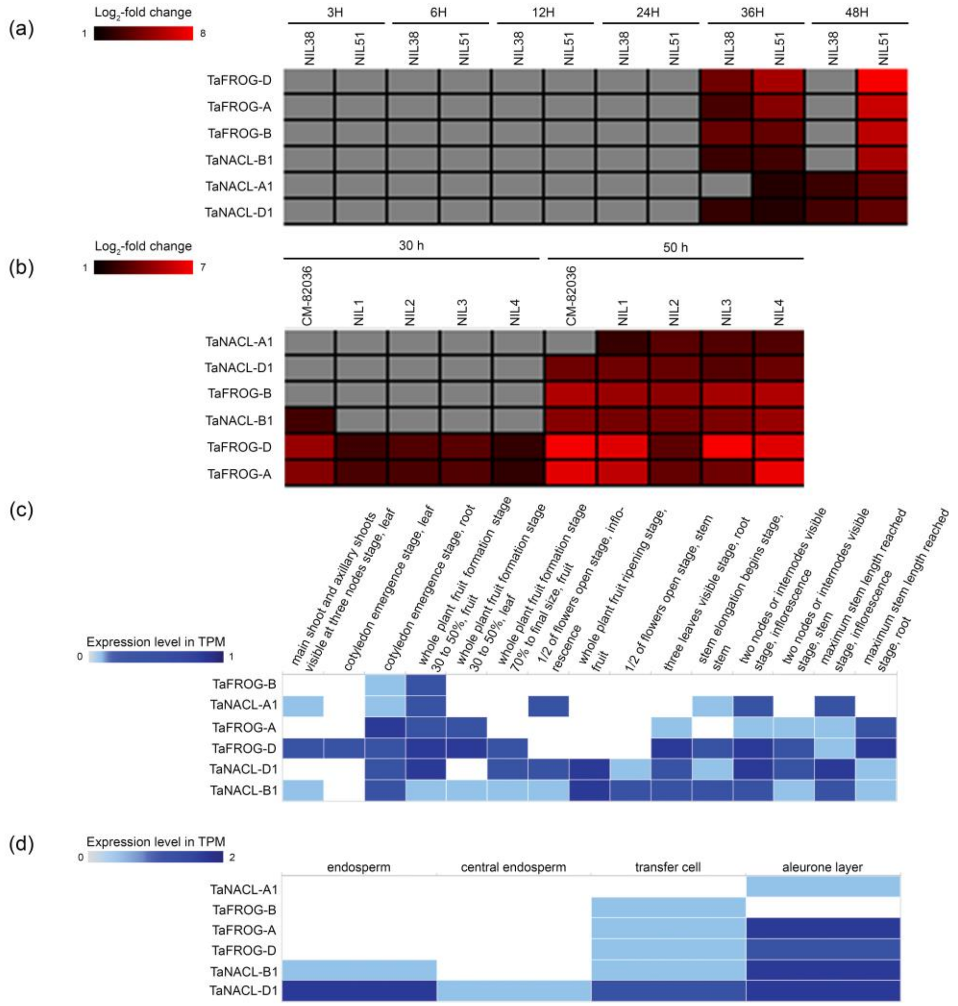

Figure S5. Expression of *TaNACL-D1* and *TaFROG* homeologs in different tissues and in response to *F. graminearum*. (a, b) Time course expression profiling of *TaNACL-D1* and *TaFROG* homeologs in wheat spikes in response to *F. graminearum*. *TaNACL-A1* (chromosome 5A homeolog), *TaNACL-B1* (chromosome 5B homeolog), *TaNACL-D1* (chromosome 5D homeolog), *TaFROG-A* (chromosome 4A homeolog), *TaFROG-B* (chromosome 4B homeolog) and *TaFROG-D* (chromosome 4D homeolog) differential gene expression were extracted from Expression Atlas, visualised with Genesis (Sturn *et al.*, 2002), and correspond to the experiments of (a) E-MTAB-4222 and (b) E-MTAB-1729. Wheat (*Triticum aestivum*) genotypes investigated in experiment (a) are NIL38 (harboring resistance QTL, *Fhb1* and *Qfhs.ifa-5A*) and NIL51 (no QTL) or in experiment (b), CM-82036, NIL1 (harboring resistance QTL *Fhb1* and *Qfhs.ifa-5A*), NIL2 (*Fhb1*), NIL3 (*Qhfs.ifa-5A*) and NIL4 (no QTL). Only significant log_2-fold change is indicated with a cut-off of 1 and adjusted *P*-value <0.05. Greater red colour saturation means higher log_2-fold change value. (c, d), Expression level of *TaNACL-D1* in normal condition at different developmental stages and tissues. TPM (Transcripts Per kilobase Million) values representing the level of expression are indicated. Data were extracted from Expression Atlas and correspond to the experiments E-MTAB-4484 (c) and E-MTAB-2137 (d). Greater blue colour saturation means higher expression. TGACv1 Gene ID used to query Expression Atlas are: TRIAE_CS42_4AL_TGACv1_290177_AA0982620 (*TaFROG-A*), TRIAE_CS42_4BS_TGACv1_328726_AA1092690 (*TaFROG-B*), TRIAE_CS42_4DS_TGACv1_361061_AA1159970 (*TaFROG-D*), TRIAE_CS42_5AS_TGACv1_392783_AA1264600 (*TaNACL-A1*), TRIAE_CS42_5BS_TGACv1_423508_AA1378340 (*TaNACL-B1*) and TRIAE_CS42_5DS_TGACv1_457805_AA1489940 (*TaNACL-D1*).

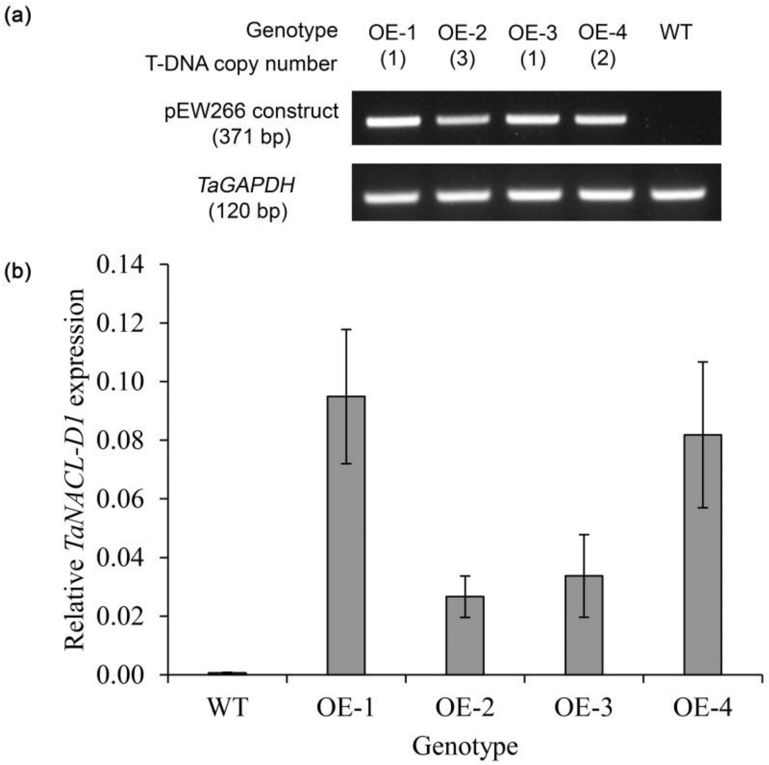

Figure S6. Molecular characterization of transgenic wheat (*Triticum aestivum*) cv Fielder overexpressing *TaNACL-D1*. (a) Verification by PCR of the T-DNA insertion in *TaNACL-D1* transgenic lines OE-1, OE-2, OE-3 and OE-4 using primers targeting the pEW266-TaNACL-D1 construct. The wild type plant (WT) and a pair of primers targeting the endogenous gene *TaGAPDH* were used as PCR negative and positive controls, respectively. For each line, the T-DNA copy number was determined using a qPCR assay (Milner *et al.*, 2018) and results are indicated in parenthesis. (b) The transcript level of *TaNACL-D1* in transgenic lines and wild type plants grown under normal plant growth conditions was determined by qRT-PCR. Error bars indicate ± SEM (n = 6).

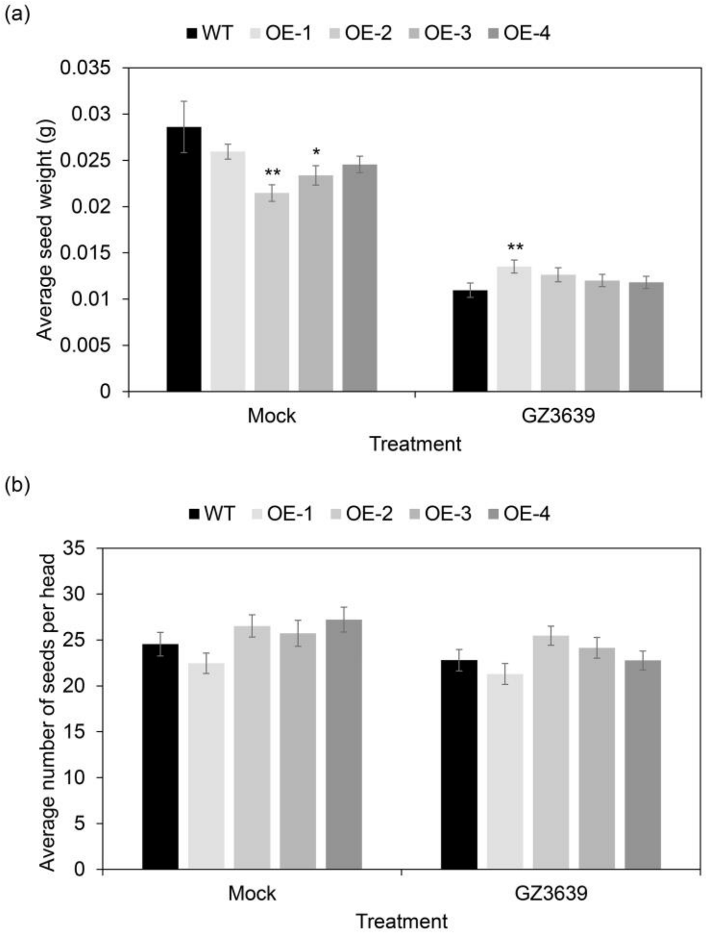

Figure S7. Effect of TaNACL-D1 overexpression on (a) average seed weight and (b) average number of seeds per head in mock and *F. graminearum* (strain GZ3639)-inoculated wheat. Results represent the mean of four trials (each has a minimum of 15 heads per genotype subjected to each treatment) and error bars indicate ± SEM (a, b: n = 80-84). Asterisks show significant differences compared to the WT (Mann-Whitney *U* test; *, *P*<0.05).

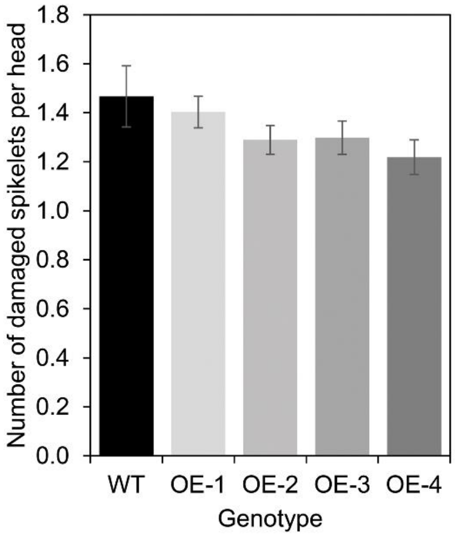

Figure S8. Effect of *TaNACL-D1* overexpression on DON tolerance. Central flowering spikelets were treated with either 16.87 mM DON or Tween-20 (mock). The phenotypic response of control plants (WT) and *TaNACL-D1* overexpression lines (OE-1, OE-2, OE-3 and OE-4) was assessed at 21 d post-toxin treatment. Results represent the mean of four trials (within each, 15 – 22 heads per genotype were subjected to each treatment) and error bars indicate ± SEM (a, b: n = 61-74).

Table S1. Primer sets used in this study.

Primers	Primer sequences (5' to 3')	Application
TaNACL-D1 ATG -27 for	GGAACCAAGGAACCATTCGTTTGC	Cloning *TaNACL-D1* CDS
TaNACL-D1 TGA +89 rev	GGAATGTCGAGAACCAAGGATCC	
TaNAC4 for	AGGAAACGAGCCATCGACTA	Cloning *TaNAC4* CDS
TaNAC4 rev	CAGAATCATGCCTGGGAAAT	
TaNACL-D1 M1 monocot for GWY	GGGGACAAGTTTGTACAAAAAAGC AGGCTCCACCATGGAAGCCTCGCGG TTCG	Cloning *TaNACL-D1* into pSc4ActR1R2
TaNACL-D1 M1 for GWY	GGAGATAGAACCATGGAAGCCTCG CGGTTCG	Cloning *TaNACL-D1* into pDONR207
TaNACL-D1 without Stop Rev GWY	CAAGAAAGCTGGGTCTGCCCCATAT CGCCGCCGT	Cloning *TaNACL-D1* into pDONR207
TaNACL-D1 Stop Rev GWY	CAAGAAAGCTGGGTCTCATGCCCCA TATCGCCGCCGT	Cloning *TaNACL-D1* into pDONR207 and pSc4ActR1R2
TaNACL-D1-mNLS -Stop rev GWY	CAAGAAAGCTGGGTCTGCCCCATAT GGCCCCGGTATGGGTAT	Cloning *TaNACL-D1* with mutated NLS into pDONR207
TaNACL-D1 -ΔNLS -Stop rev GWY	CAAGAAAGCTGGGTCGCTCTTGTCC TTGTAGTACTCTT	Cloning *TaNACL-D1* with truncated NLS into pDONR207
TaNAC4 M1 for GWY	GGAGATAGAACCATGATGACGGCA ATGGTG	Cloning *TaNAC4* into pDONR207
TaNAC4 +Stop rev GWY	CAAGAAAGCTGGGTCTCAGAATGGT GGCAAGATTGT	
TaNAC4 -Stop rev GWY	CAAGAAAGCTGGGTCGAATGGTGG CAAGATTGT	
attB1	GGGGACAAGTTTGTACAAAAAAGC AGGCTTCGAAGGAGATAGAACCAT G	attB extension for subcloning into pDONR207
attB2	GGGGACCACTTTGTACAAGAAAGCT GGGTC	
pDONR207 for	TCGCGTTAACGCTAGCATGGATCTC	Sequencing pDONR207
pDONR207 rev	GTAACATCAGAGATTTTGAGACAC	
TaGAPDH for	CCTTCCGTGTTCCCACTGTTG	Control DNA contamination in RNA samples
TaGAPDH rev	ATGCCCTTGAGGTTTCCCTC	
pEW246 NOS rev	CCGCCCGATCTAGTATCATA	Confirmation T-DNA insertion
TaNACL-D1 OE for	GAACATCAACGGGTTTCACC	qRT-PCR (overexpressor lines) of *TaNACL-D1*
TaNACL-D1 OE rev	AGAAGCGGAACCGTGTAGTG	
TaNACL-D1 for	GACAAGAGCCGCGTCAAAC	qRT-PCR and confirmation T-DNA insertion with *TaNACL-D1* for
TaNACL-D1 rev	GTTCGATCAGGATGGGTCATG	
TaPP2AA3 for	TCGGAGAGTTCGTTCTAGCC	qRT-PCR housekeeping genes
TaPP2AA3 rev	CACCATCGAAACAACGTGTC	
TaYLS8 for	CAACTGGGCAATGAAAGACA	
TaYLS8 rev	CATGAATAGCCAGGCACAGA	

Supporting Information References:

Fox, S.E., Geniza, M., Hanumappa, M., Naithani, S., Sullivan, C., Preece, J., Tiwari, V.K., Elser, J., Leonard, J.M., Sage, A., Gresham, C., Kerhornou, A., Bolser, D., McCarthy, F., Kersey, P., Lazo, G.R. and Jaiswal, P. (2014) De novo transcriptome assembly and analyses of gene expression during photomorphogenesis in diploid wheat Triticum monococcum. *PLoS One* **9**, e96855.

Sturn, A., Quackenbush, J. and Trajanoski, Z. (2002) Genesis: cluster analysis of microarray data. *Bioinformatics* **18**, 207-208.
